# Supplementary material for: Total synthesis of teixobactin
Source: Nat Commun. 2016 Aug 3;7:12394. doi: 10.1038/ncomms12394 (PMC4976201; doi:10.1038/ncomms12394)
Supplement: Supplementary Information — Supplementary Figures 1-43, Supplementary Tables 1 - 2, Supplementary Methods and Supplementary References [file ncomms12394-s1.pdf]

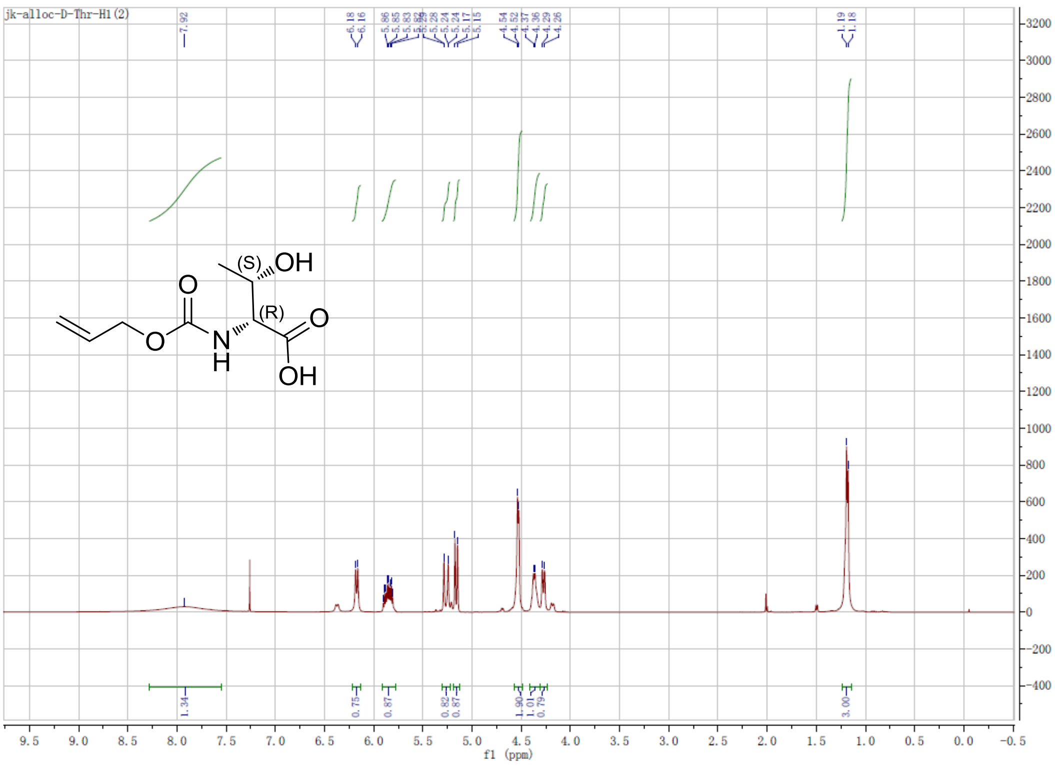

Supplementary Figure 1. ^{1}H NMR spectrum of compound 1a.

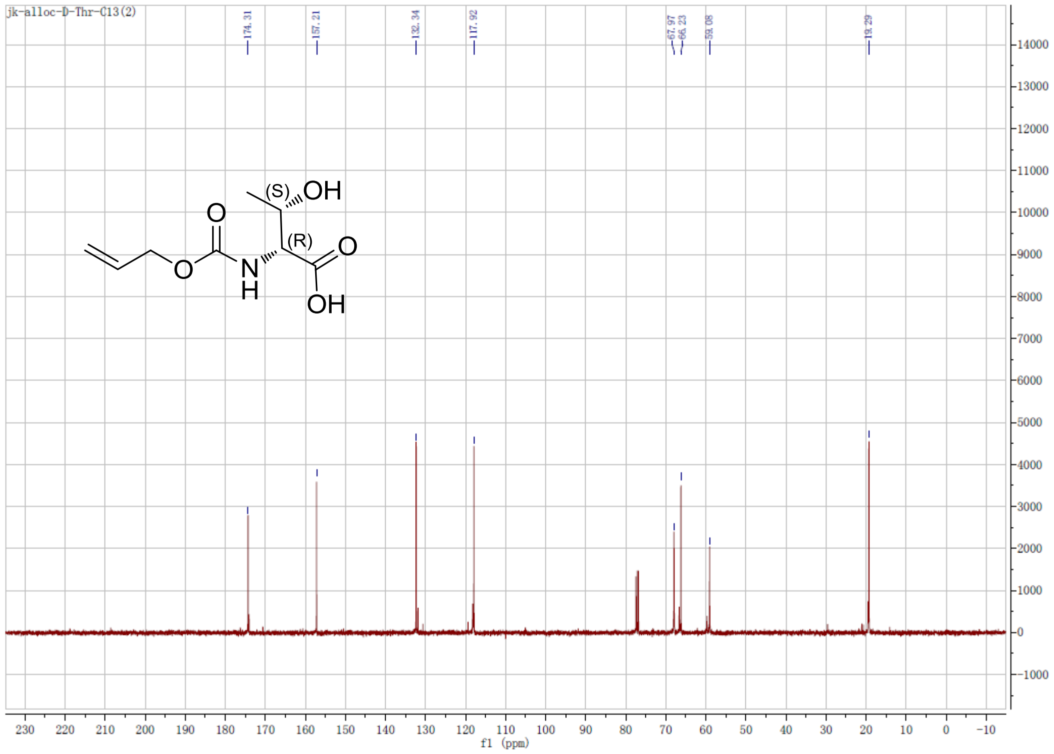

Supplementary Figure 2. ^{13}C NMR spectrum of compound 1a.

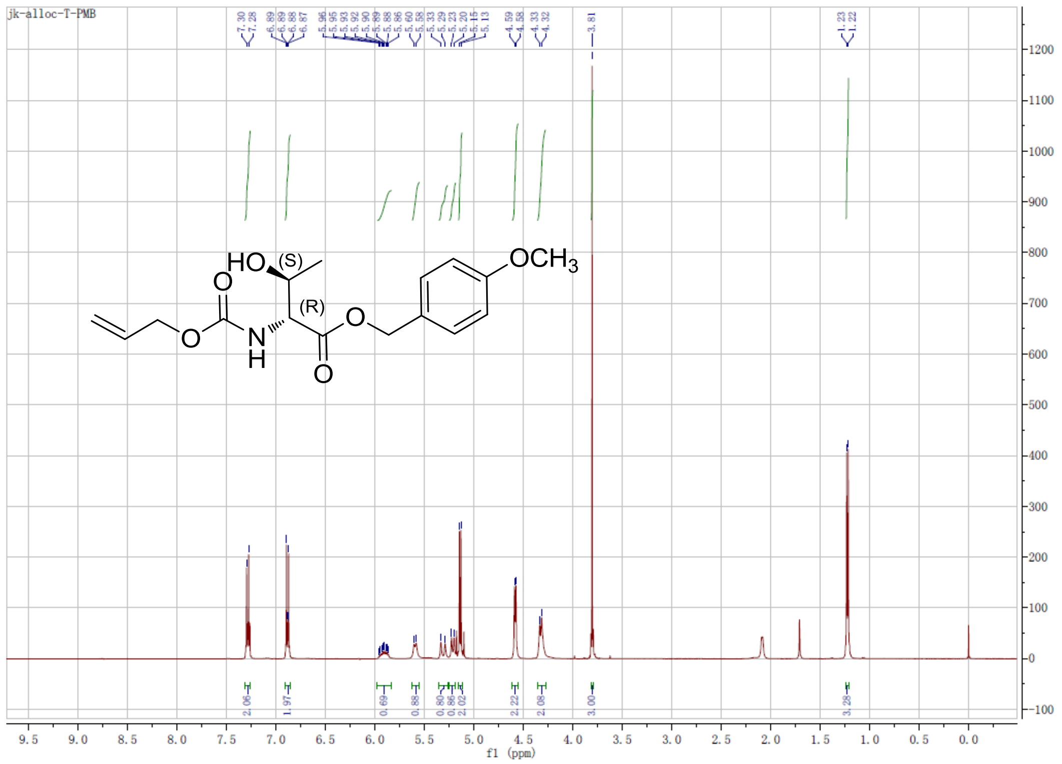

Supplementary Figure 3. ^{1}H NMR spectrum of compound 2a.

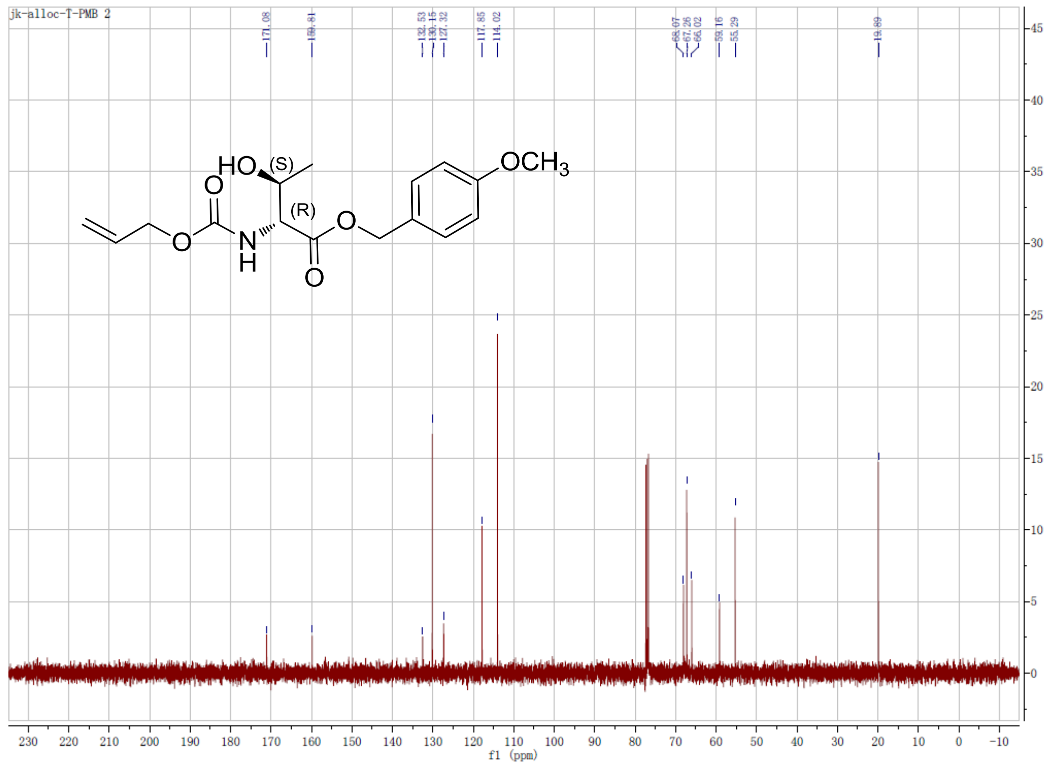

Supplementary Figure 4. ^{13}C NMR spectrum of compound 2a.

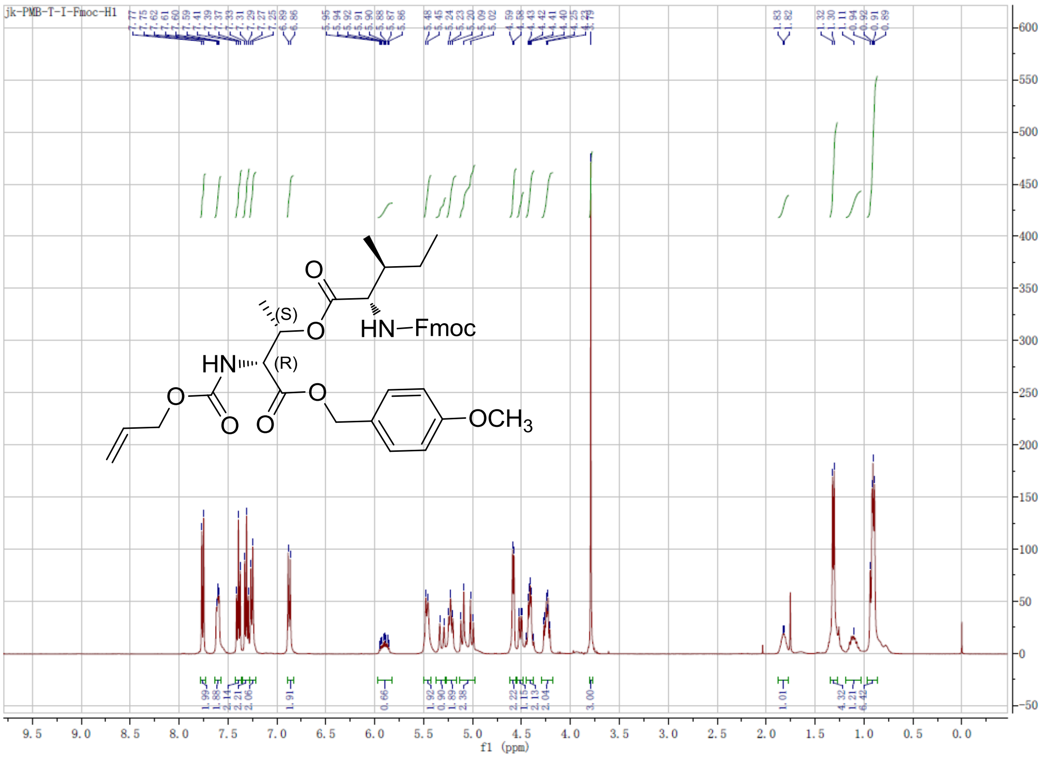

Supplementary Figure 5. ^{1}H NMR spectrum of compound 3a.

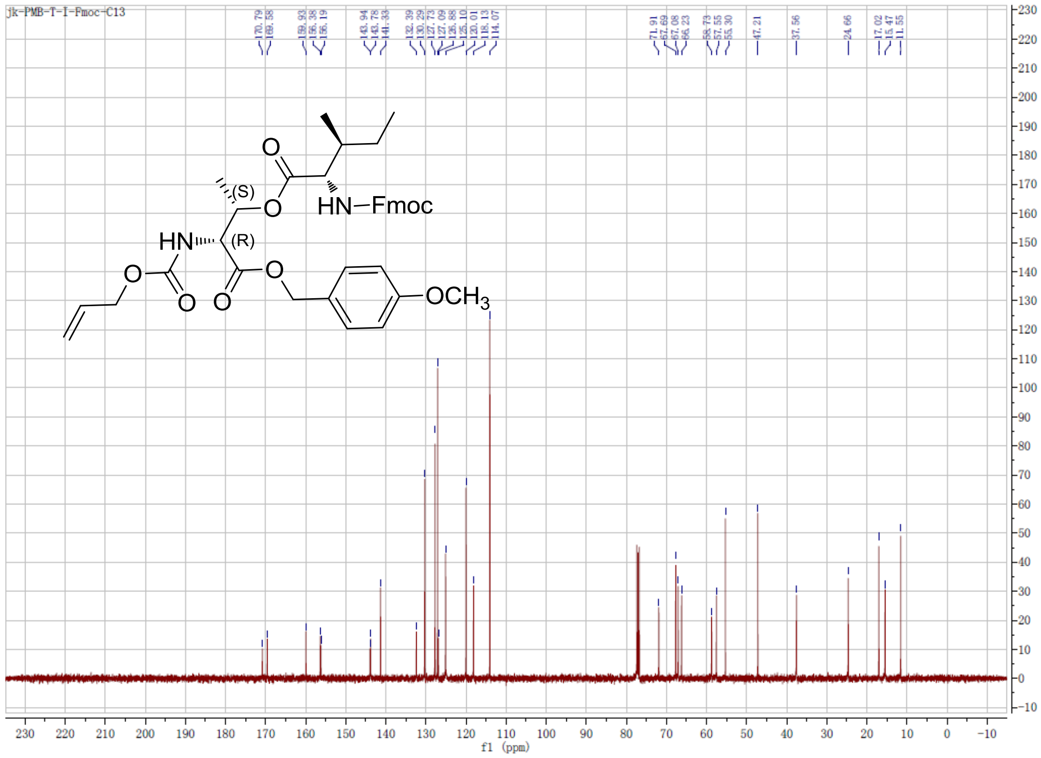

Supplementary Figure 6. ^{13}C NMR spectrum of compound 3a.

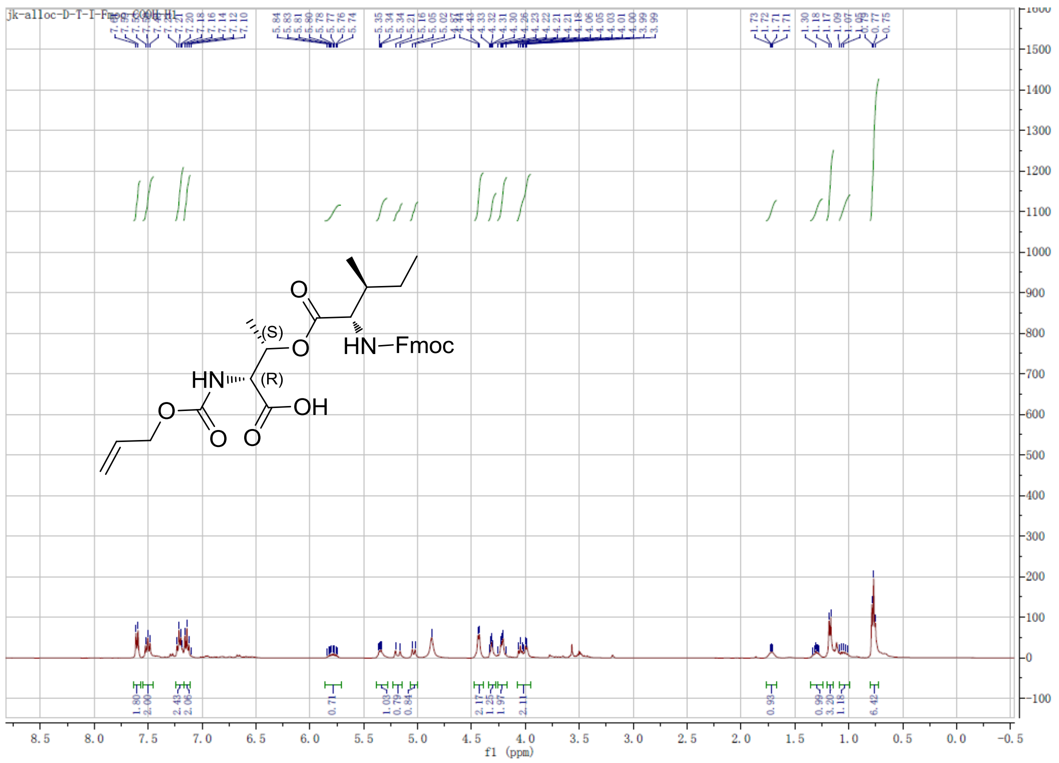

Supplementary Figure 7. ^{1}H NMR spectrum of compound 4a.

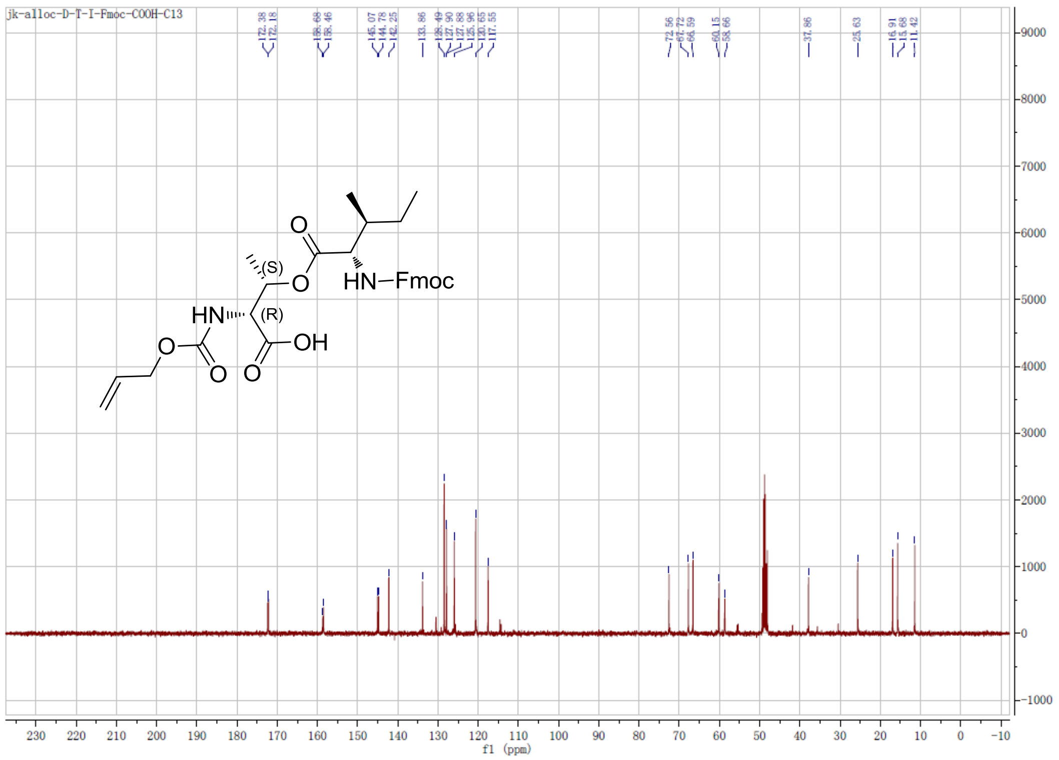

Supplementary Figure 8. ^{13}C NMR spectrum of compound 4a.

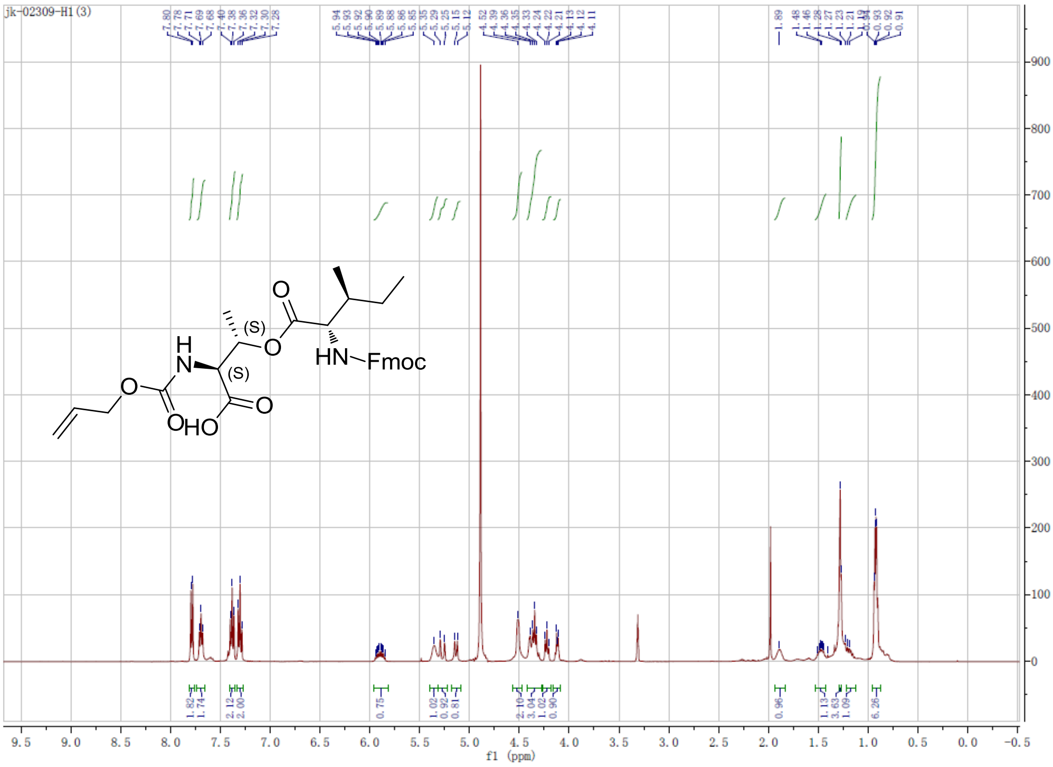

Supplementary Figure 9. ^{1}H NMR spectrum of compound 4b.

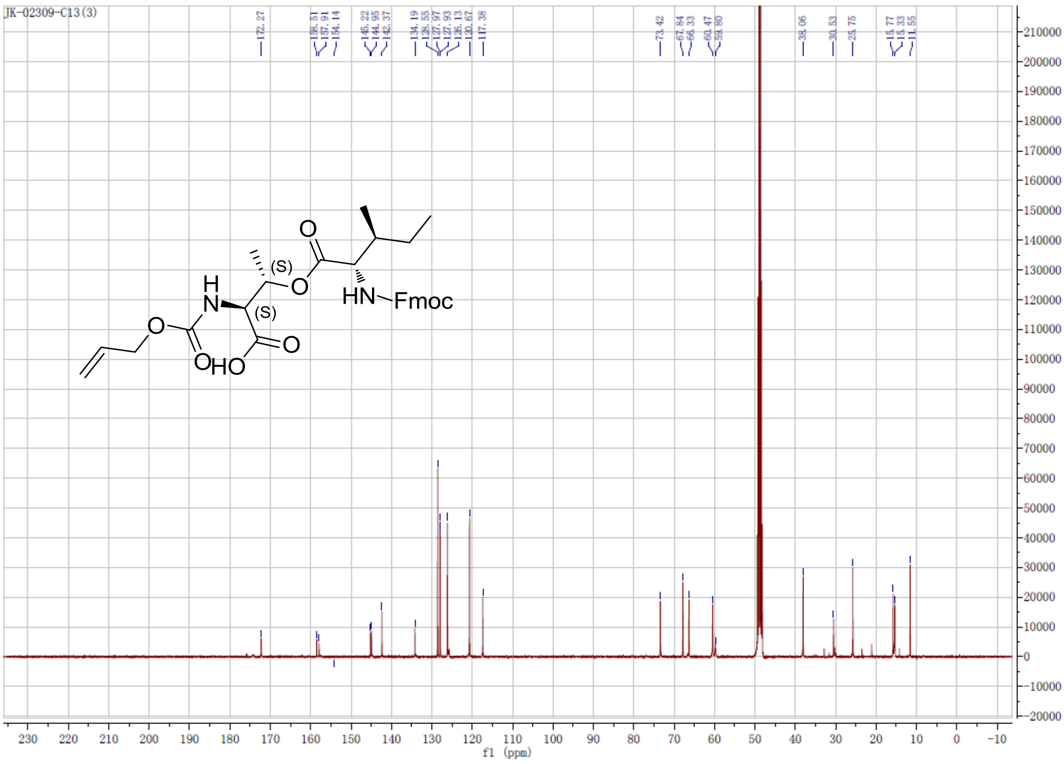

Supplementary Figure 10. ^{13}C NMR spectrum of compound 4b.

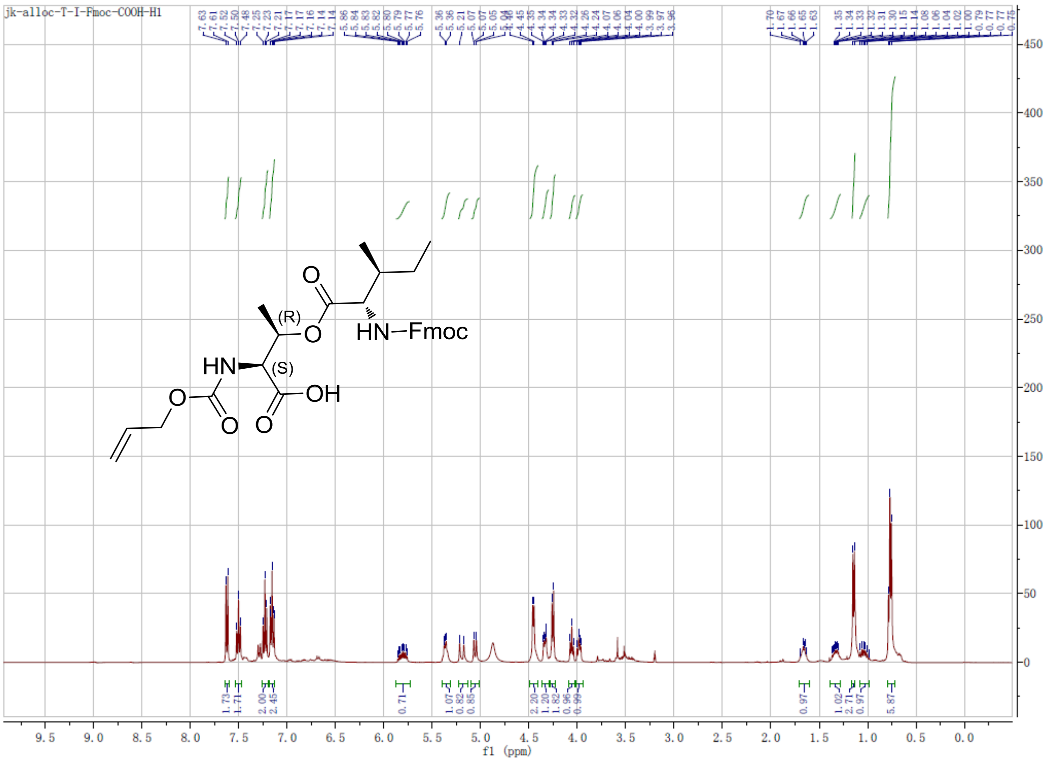

Supplementary Figure 11. ^{1}H NMR spectrum of compound 4c.

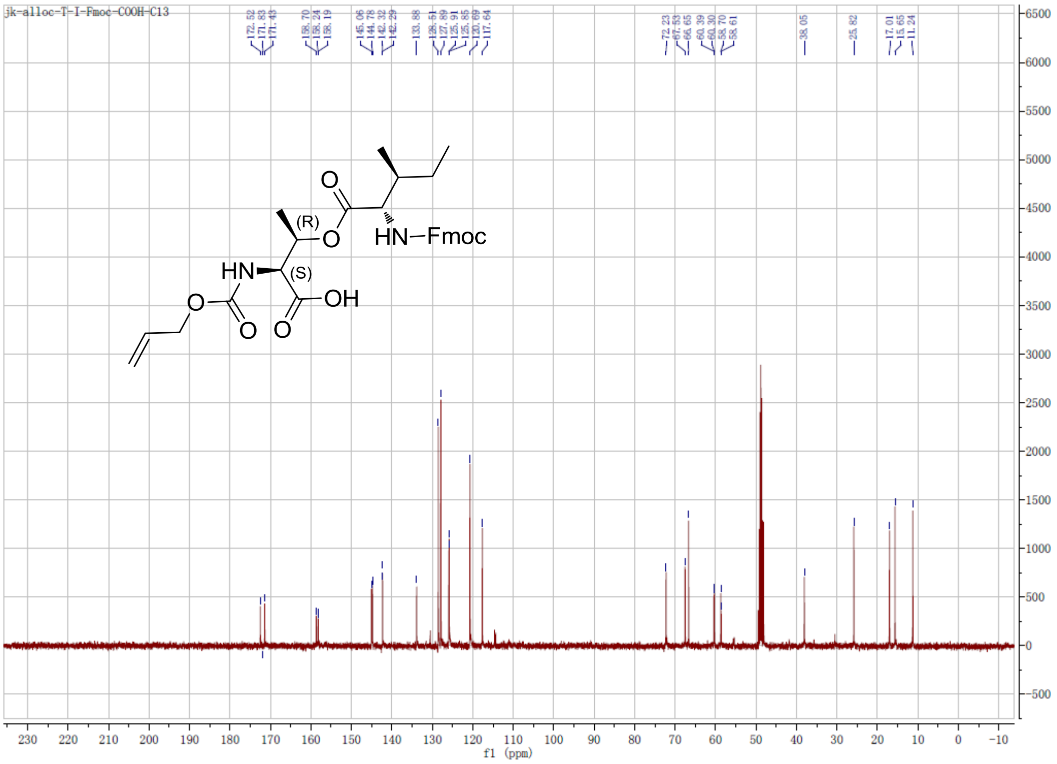

Supplementary Figure 12. ^{13}C NMR spectrum of compound 4c.

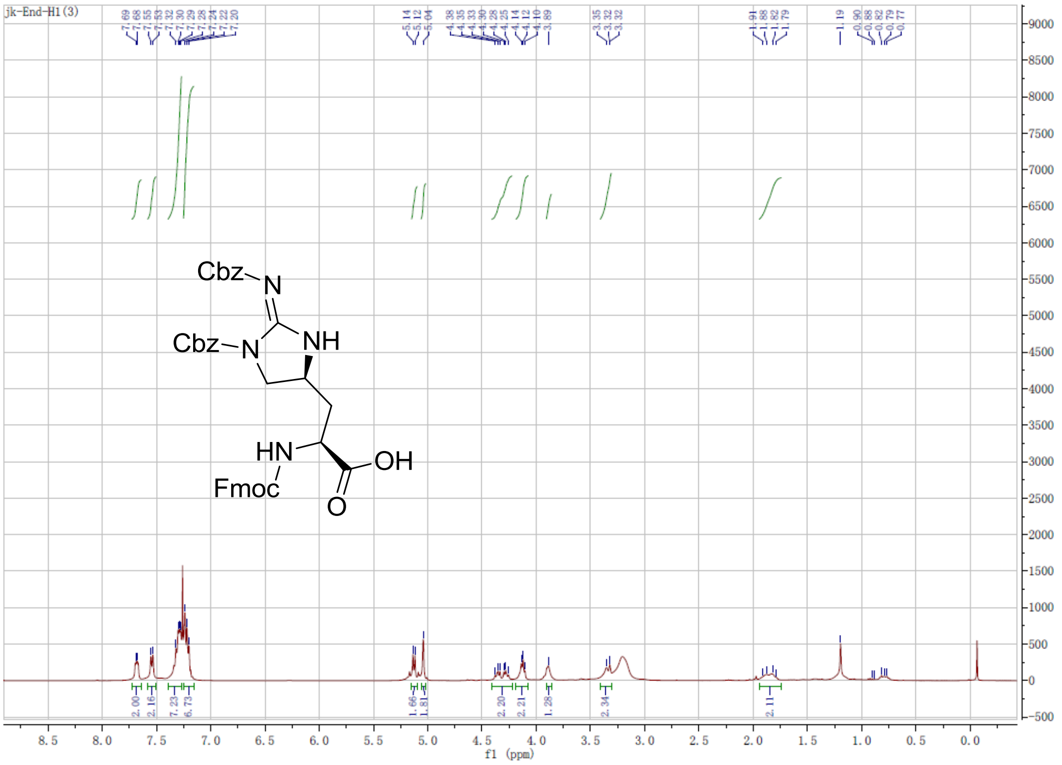

Supplementary Figure 13. ^{1}H NMR spectrum of compound S2.

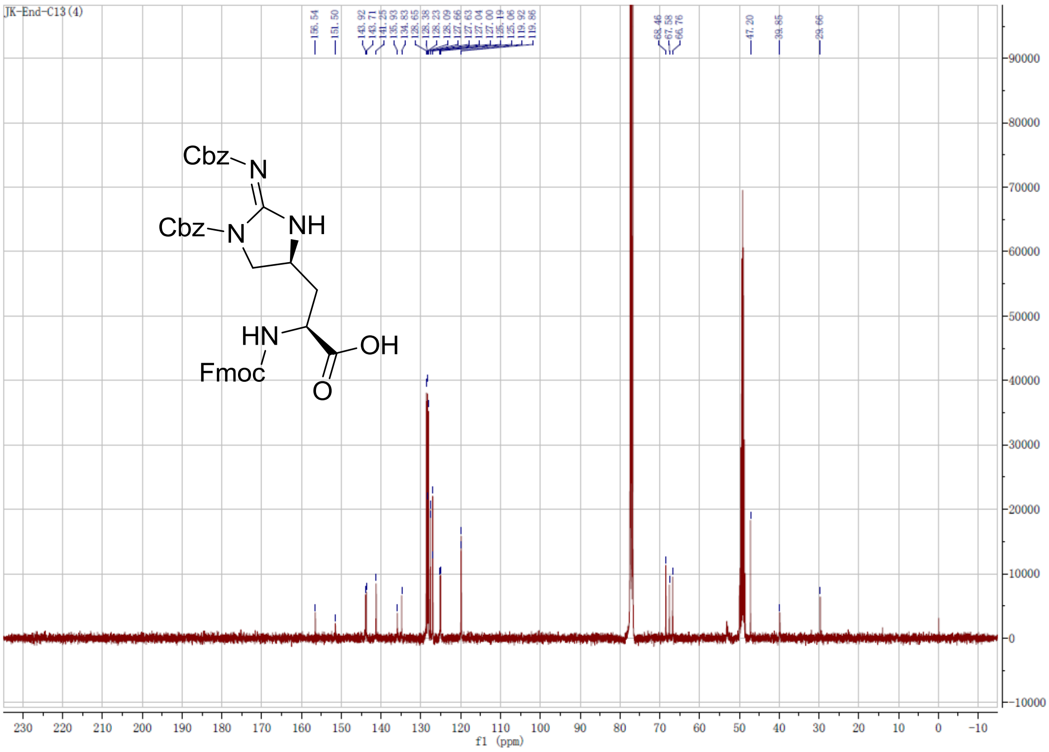

Supplementary Figure 14. ^{13}C NMR spectrum of compound S2.

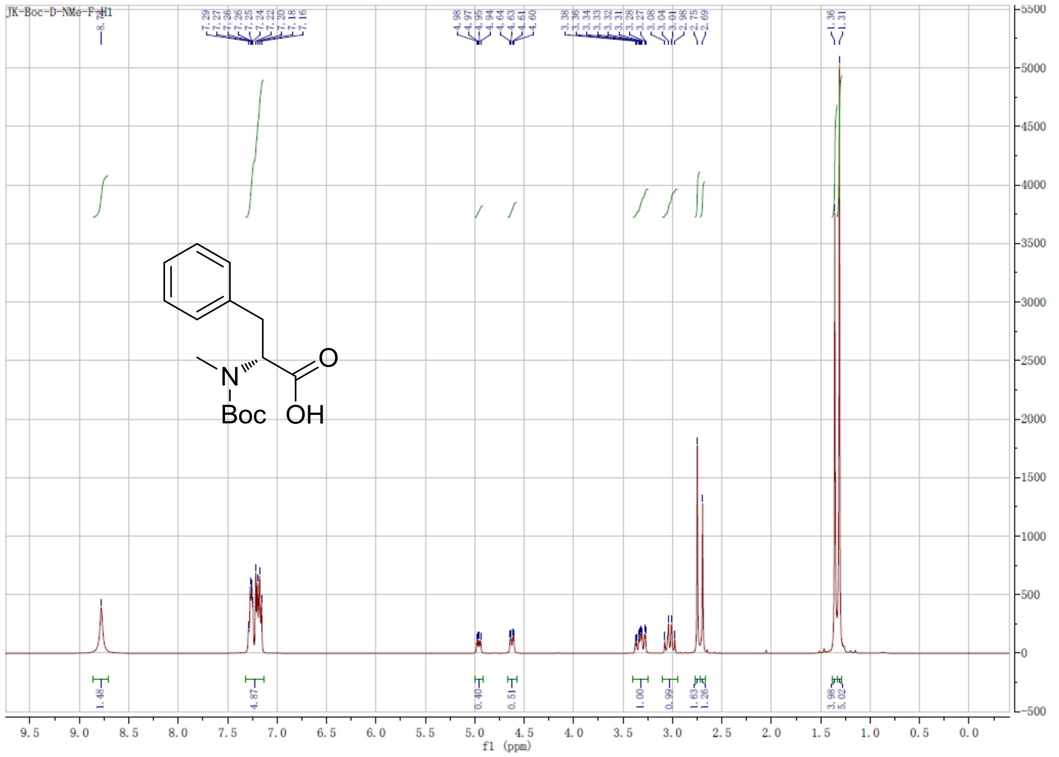

Supplementary Figure 15. ^{1}H NMR spectrum of compound S3.

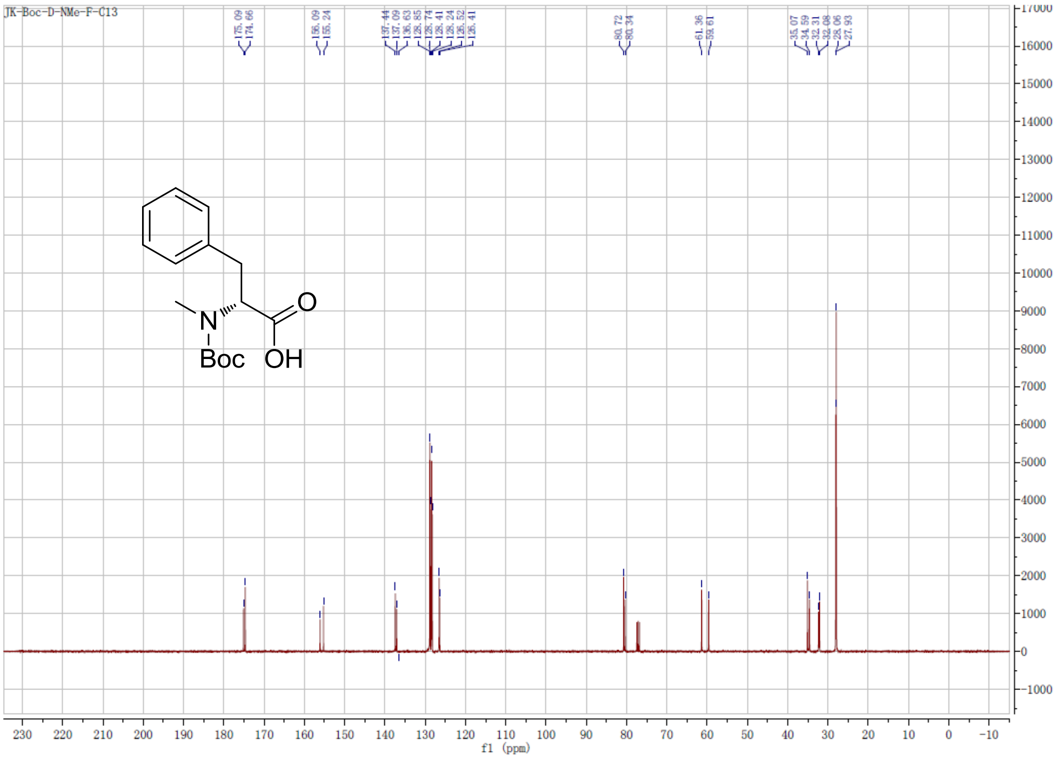

Supplementary Figure 16. ^{13}C NMR spectrum of compound S3.

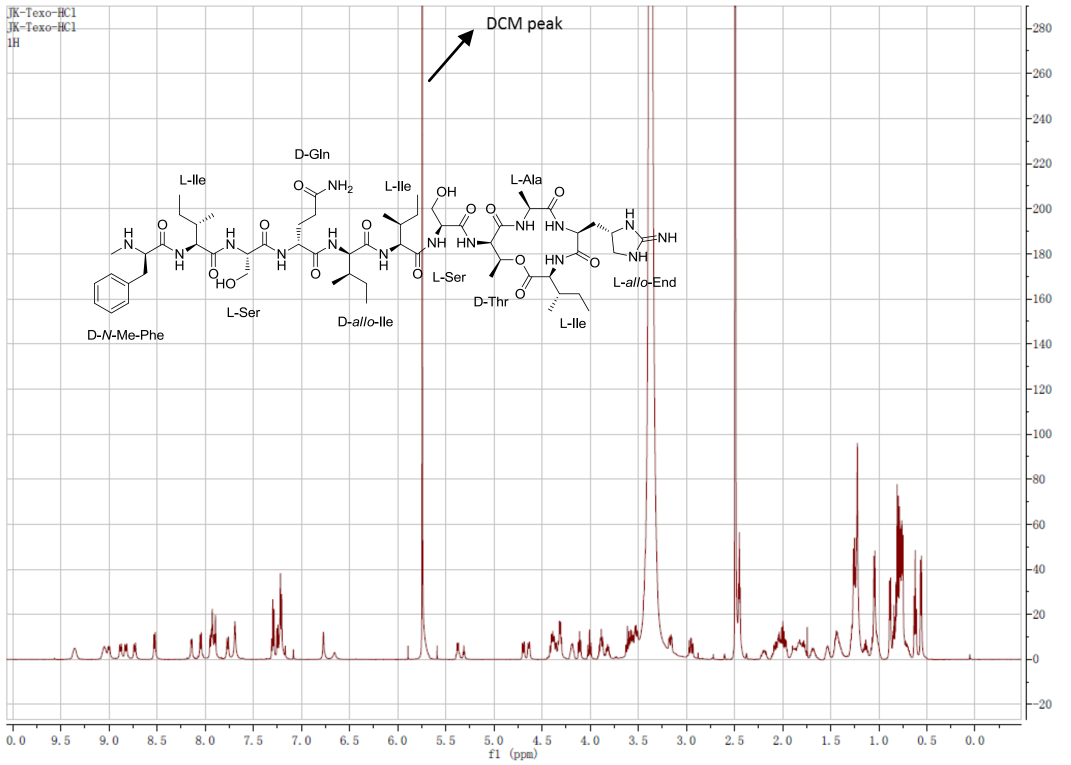

Supplementary Figure 17. ^{1}H NMR spectrum of compound 12 (HCl Salt).

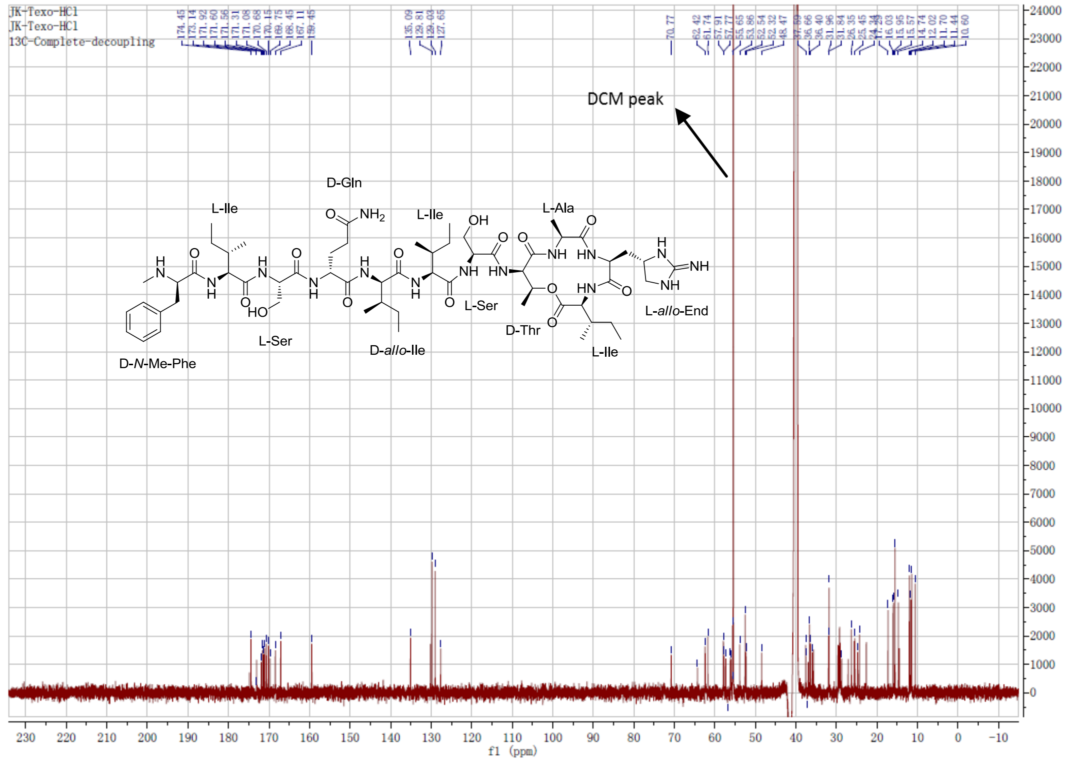

Supplementary Figure 18. ^{13}C NMR spectrum of compound 12 (HCl Salt).

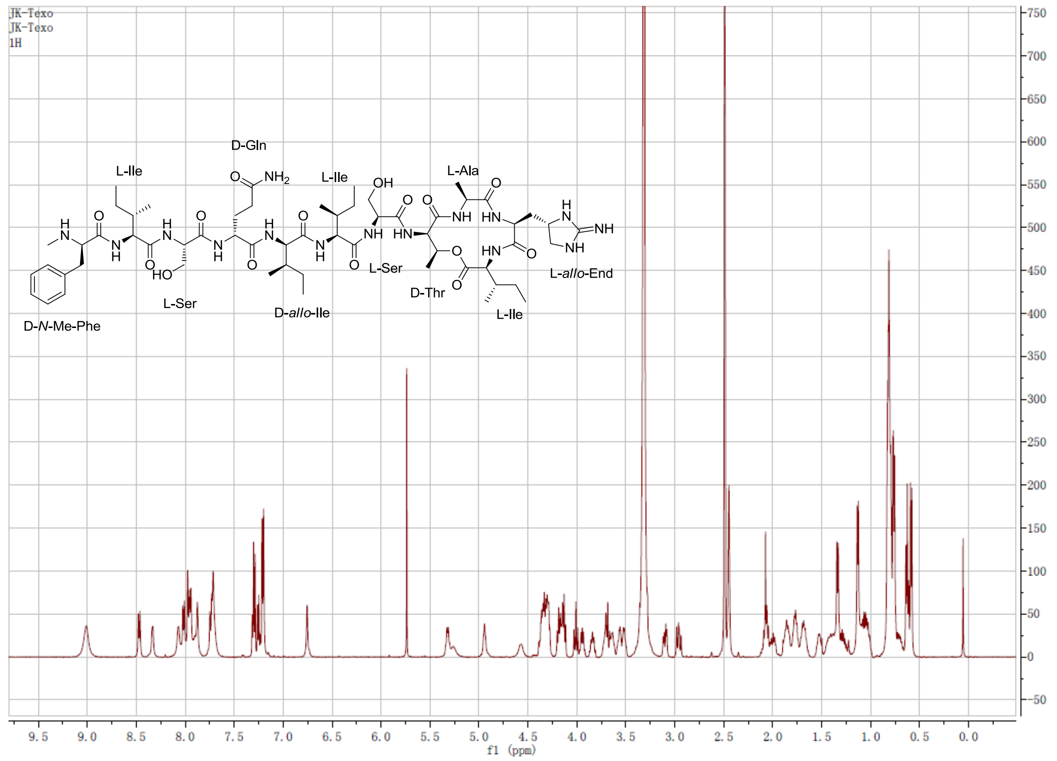

Supplementary Figure 19. ^{1}**H NMR spectrum of compound 12 (TFA Salt).**

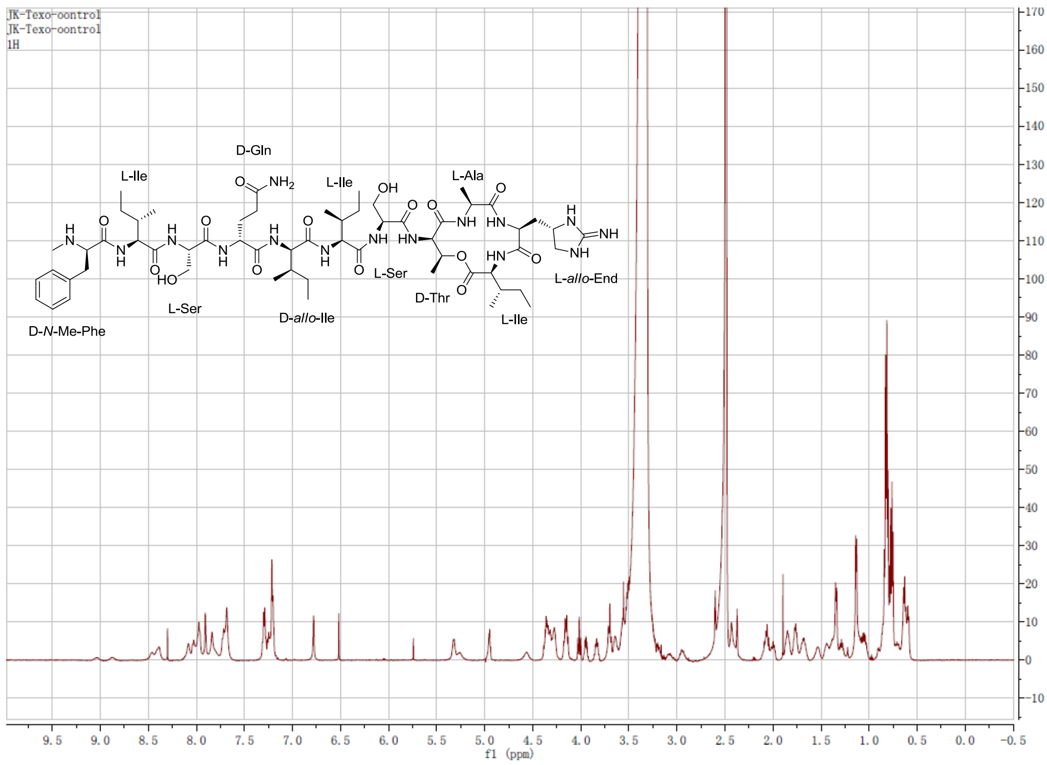

Supplementary Figure 20. ^{1}**H NMR spectrum of authentic sample of teixobactin (TFA Salt).**

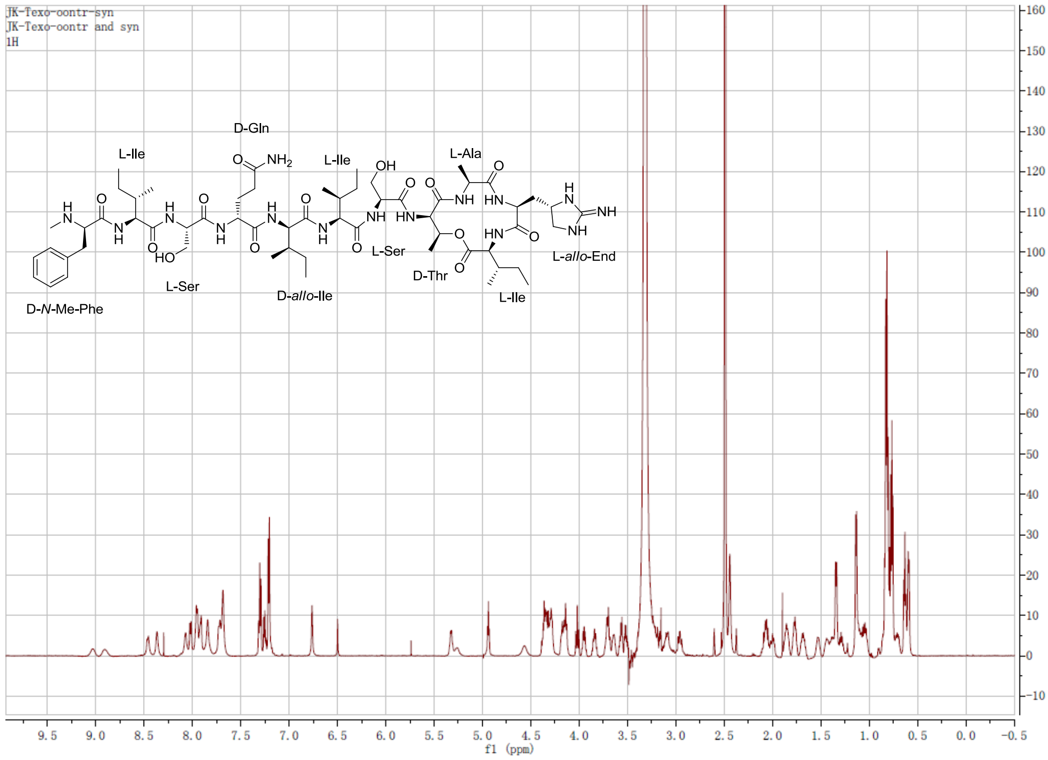

Supplementary Figure 21. ^{1}**H NMR spectrum of the mixture of authentic sample of teixobactin (80%) and compound 12 (20%) (TFA Salt).**

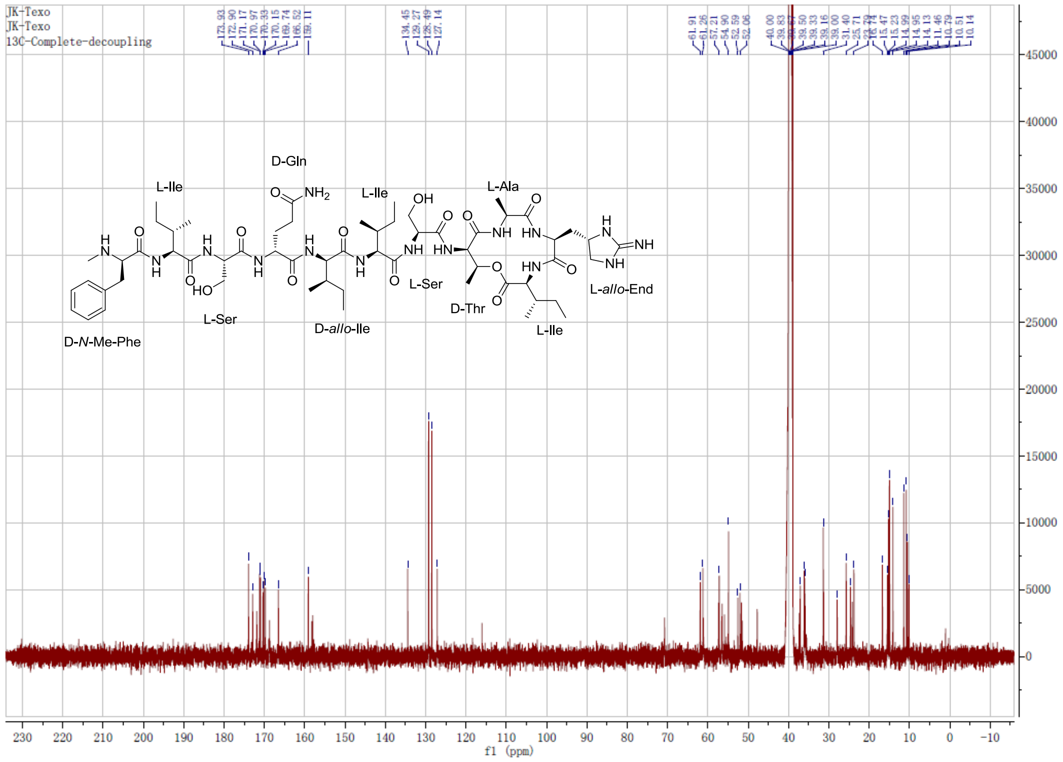

Supplementary Figure 22. ^{13}**C NMR spectrum of compound 12 (TFA Salt).**

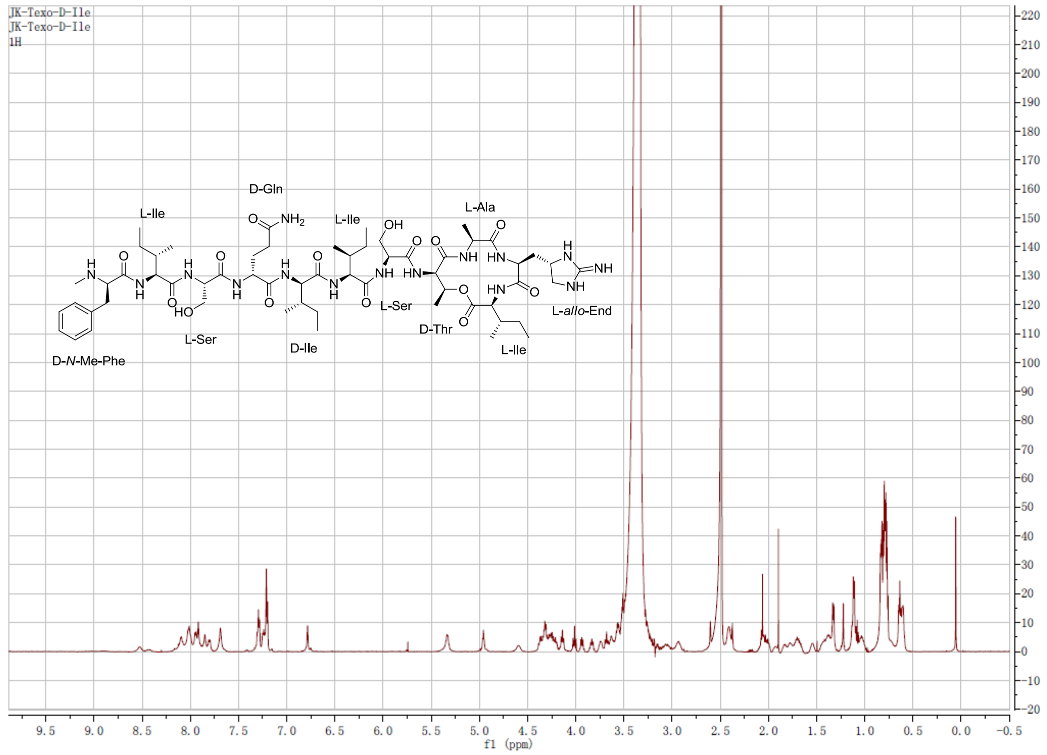

Supplementary Figure 23. ^{1}H NMR spectrum of compound 13 (TFA Salt).

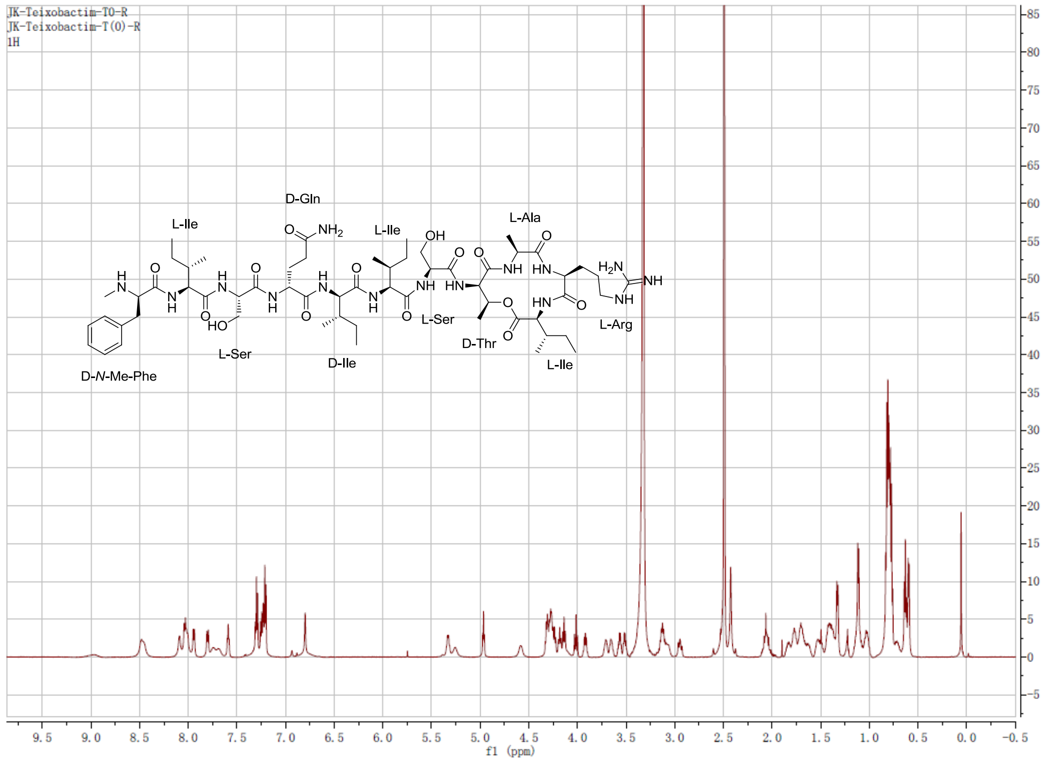

Supplementary Figure 24. ^{1}H NMR spectrum of compound 14 (TFA Salt).

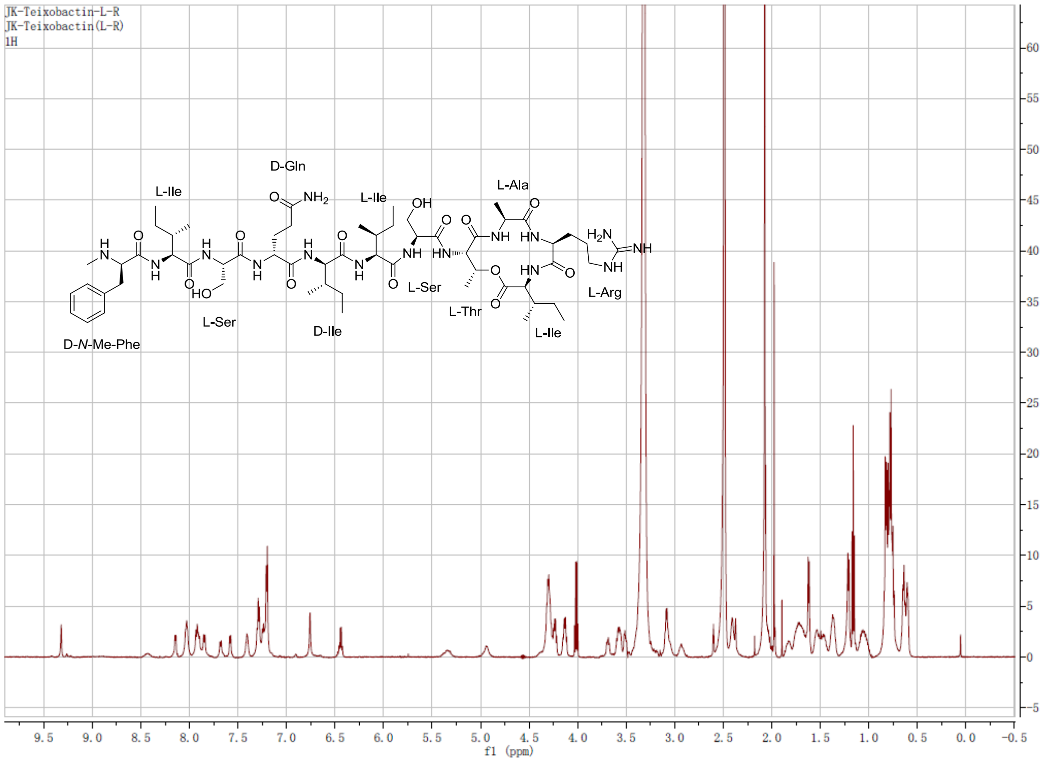

Supplementary Figure 25. ^{1}H NMR spectrum of compound 15 (TFA Salt).

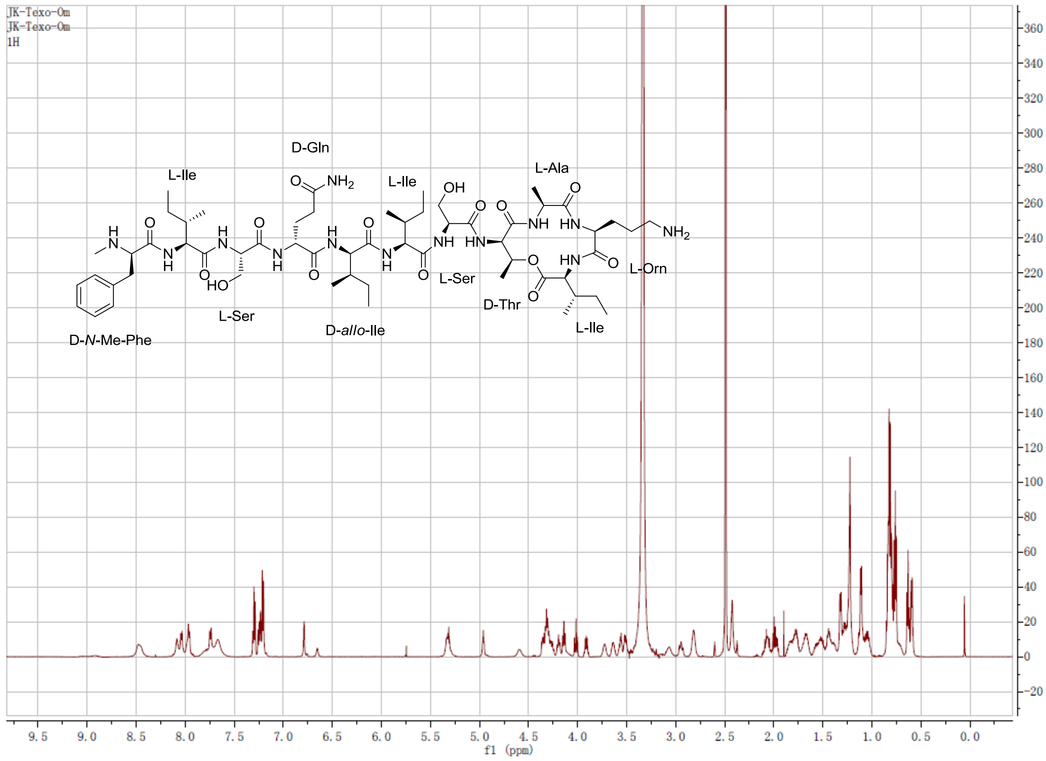

Supplementary Figure 26. ^{1}H NMR spectrum of compound 16 (TFA Salt).

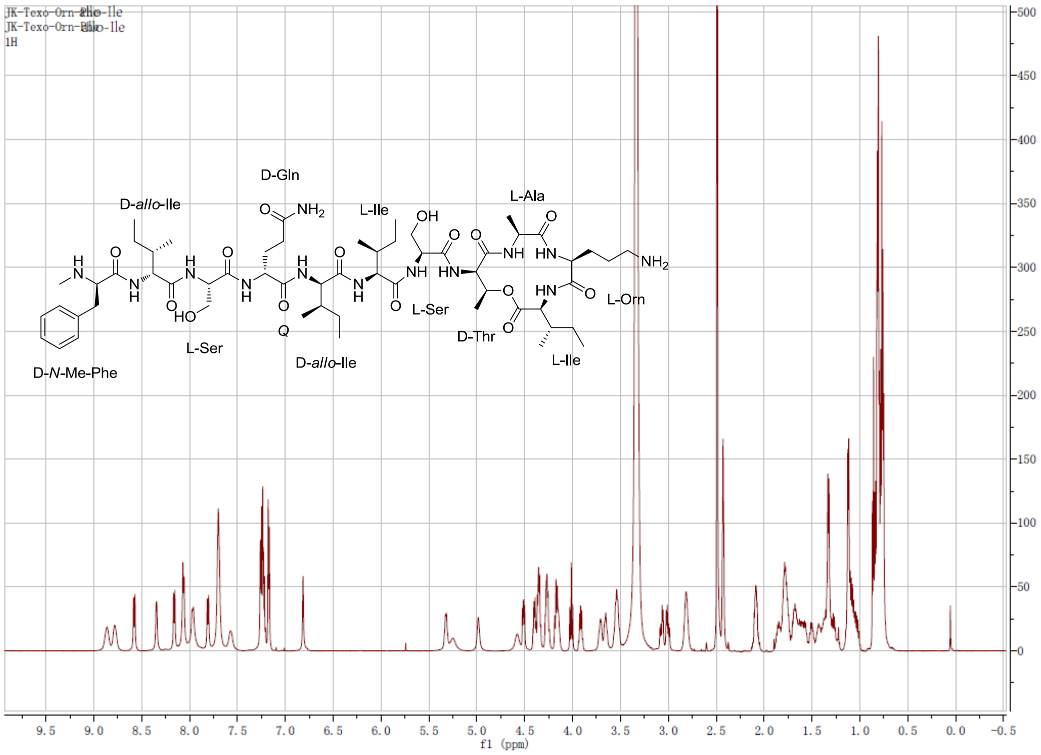

Supplementary Figure 27. ^{1}H NMR spectrum of compound 17 (TFA Salt).

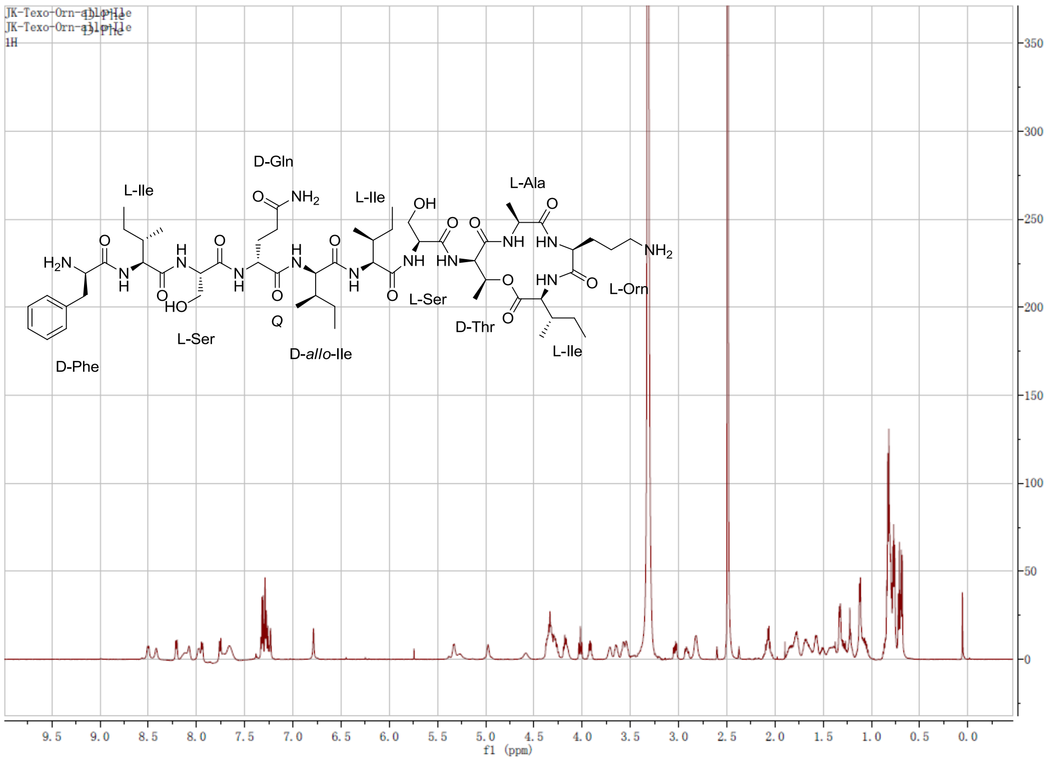

Supplementary Figure 28. ^{1}H NMR spectrum of compound 18 (TFA Salt).

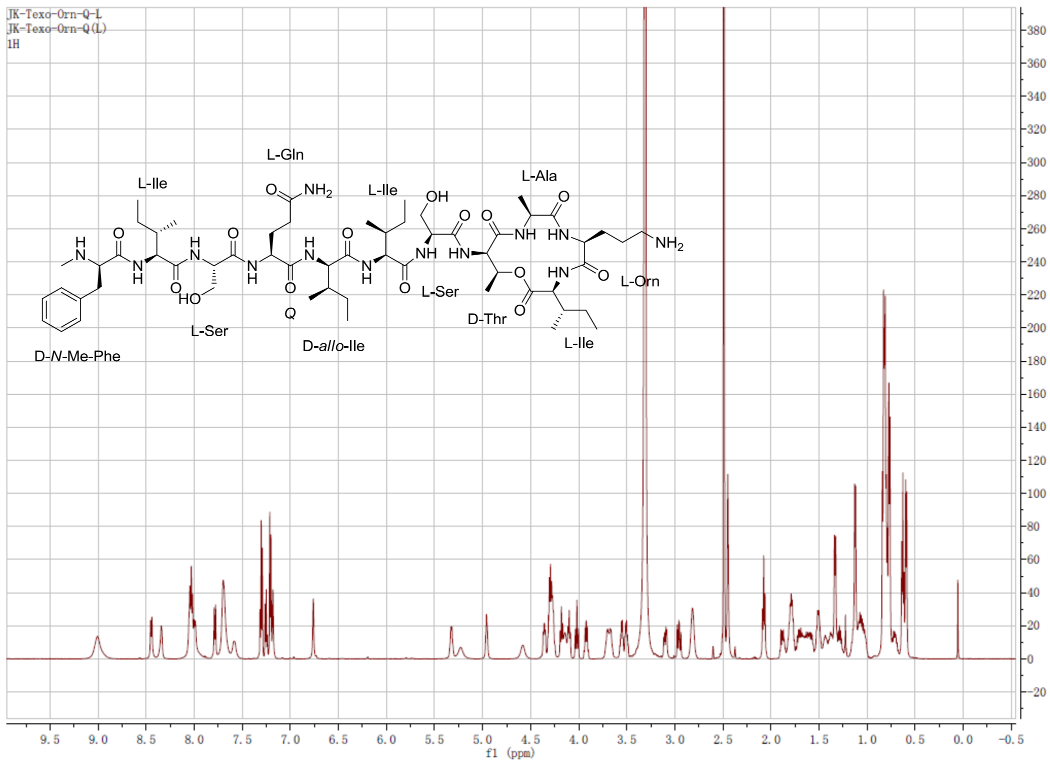

Supplementary Figure 29. ^{1}**H NMR spectrum of compound 19 (TFA Salt).**

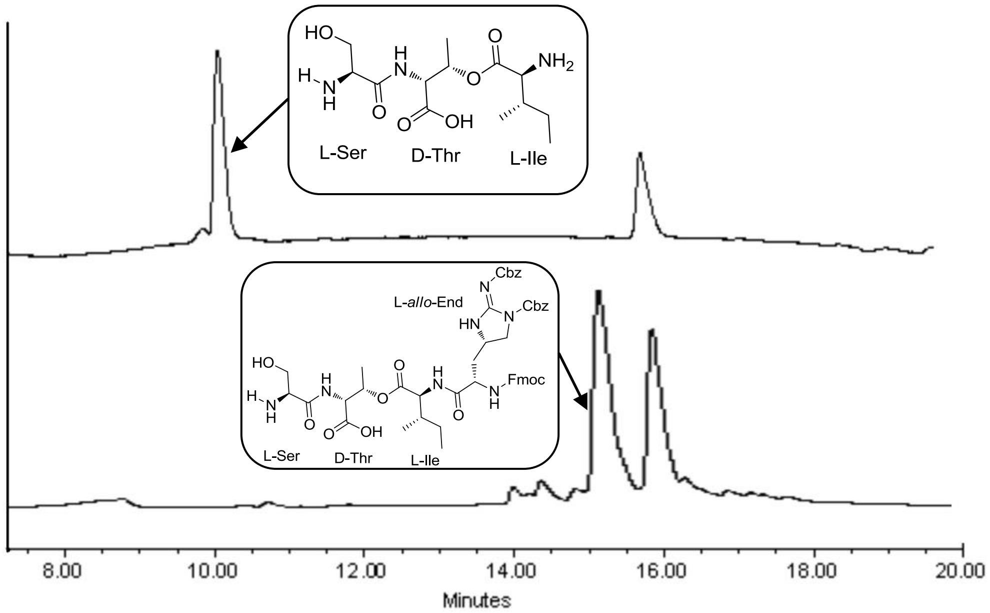

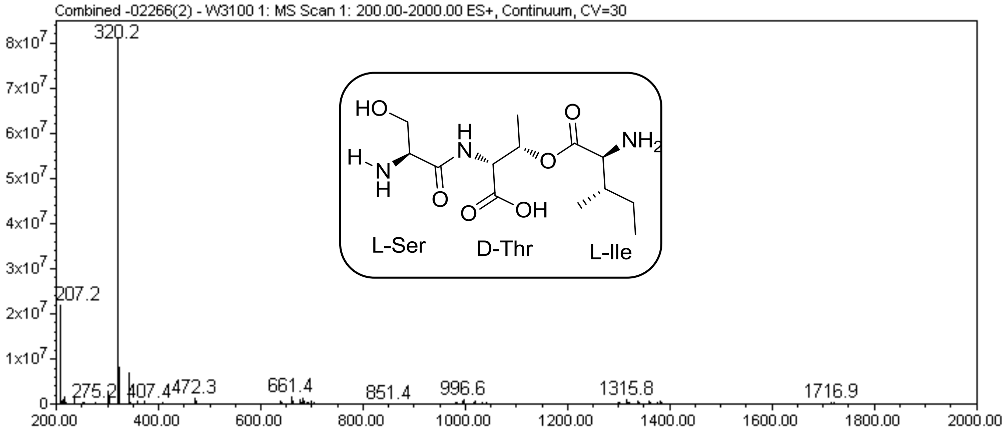

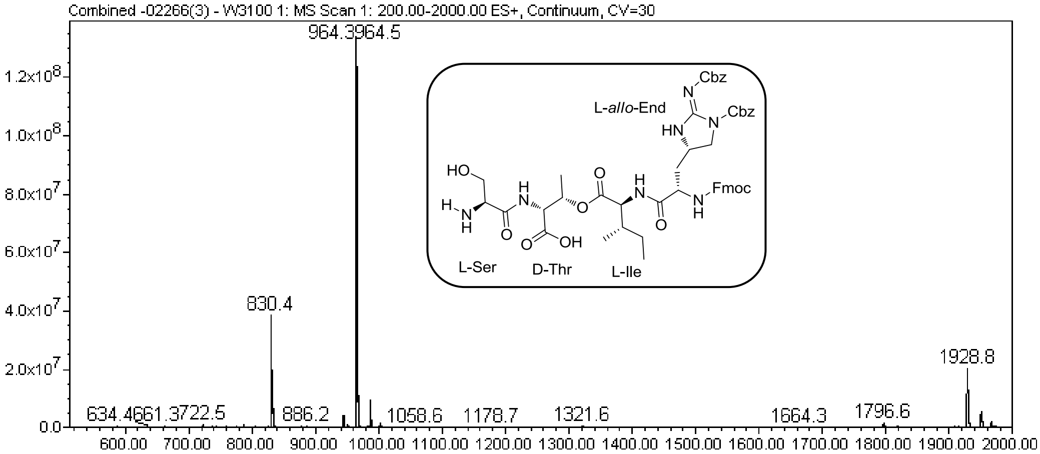

Supplementary Figure 30. UV trace and corresponding masses from LC-MS analysis of the starting material and crude product of the reaction of coupling Fmoc-L-*allo*-End(Cbz)$_2$-OH. Gradient: 5—95% CH_3CN/H_2O with 0.1% TFA over 15 min at a flow rate of 0.6 mL/min. ESI: calculated for $C_{13}H_{26}N_3O_6^+$ [M+H$^+$]: 320.2; found: 320.2; ESI: calculated for $C_{50}H_{58}N_7O_{13}^+$ [M+H$^+$]: 964.4; found: 964.4

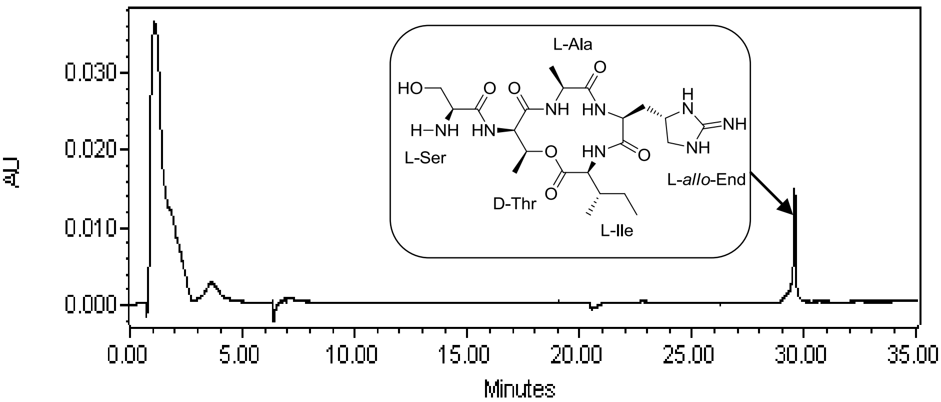

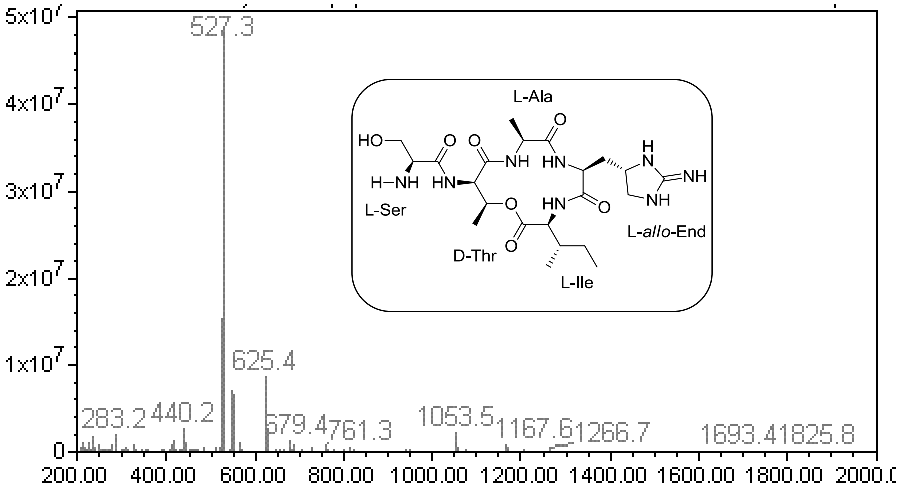

Supplementary Figure 31. UV trace and corresponding mass from LC-MS analysis of compound 9. Gradient: 5—50% CH_3CN/H_2O with 0.1% TFA over 15 min at a flow rate of 0.6 mL/min. ESI: calculated for $C_{22}H_{39}N_8O_7^+$ [M+H$^+$]: 527.3; found: 527.3

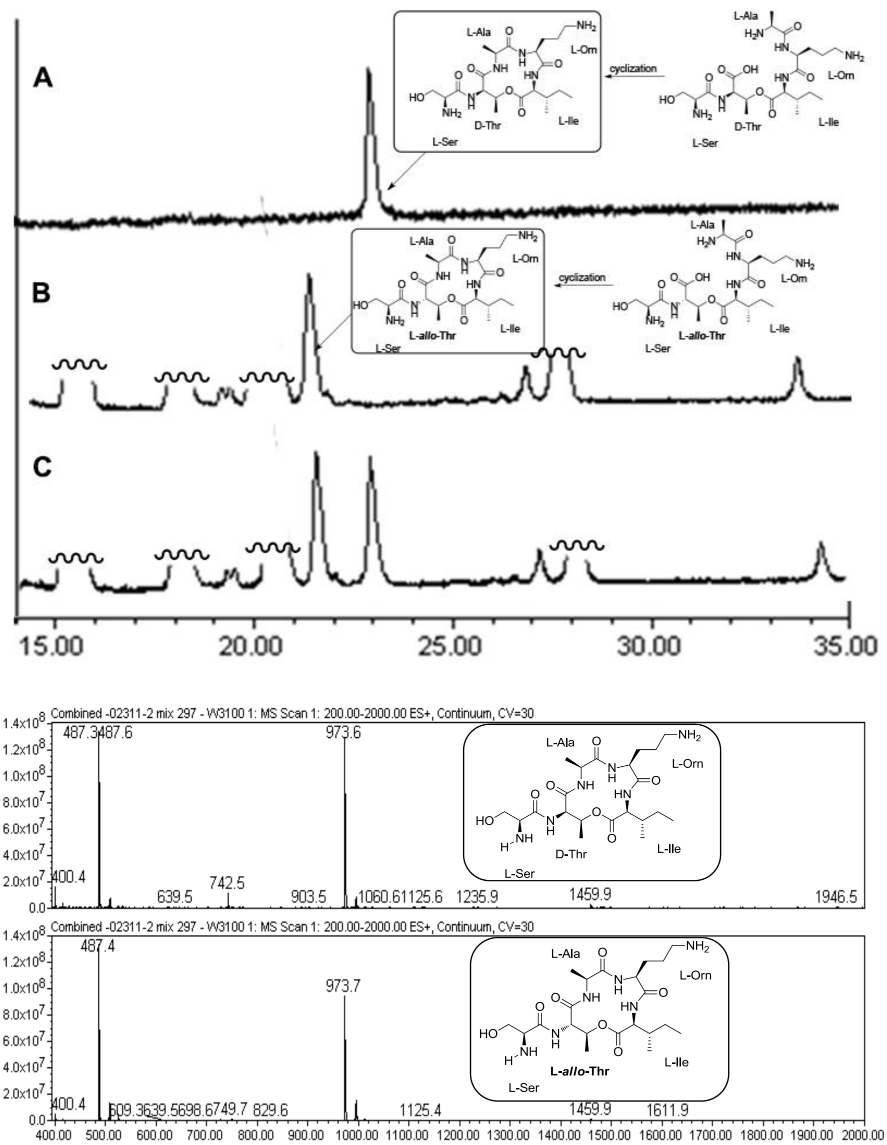

Supplementary Figure 32. UV trace and corresponding masses from LC-MS analysis of compound S4 and crude reaction mixture of compound S5. Gradient: 0—40% CH_3CN/H_2O with 0.1% TFA over 30 min at a flow rate of 0.6 mL/min. **A:** compound **S4** was purified by preparative HPLC (5-50% CH3CN/H2O over 30 min) before LC-MS analysis. **B:** crude reaction mixture of compound **S5**. **C:** co-injection of **A** and **B**. ESI: calculated for $C_{21}H_{39}N_6O_7^+$ $[M+H^+]$: 487.3; found: 487.4

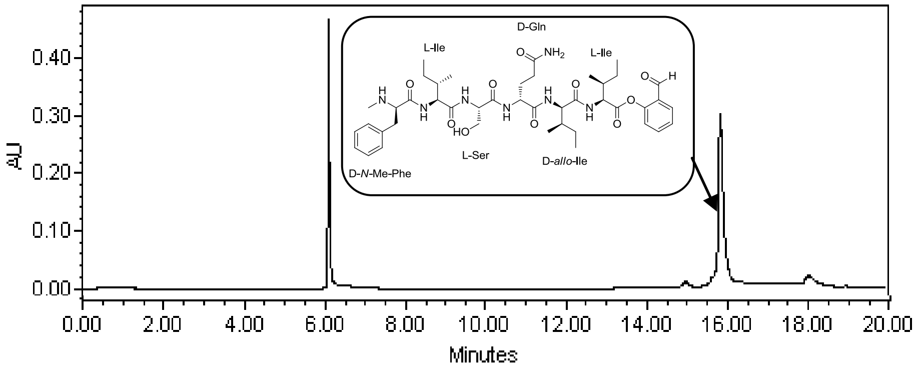

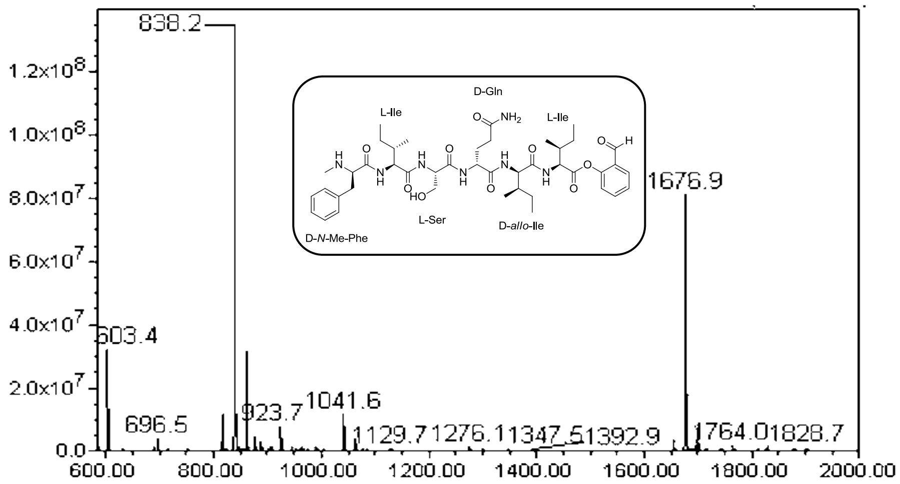

Supplementary Figure 33. UV trace and corresponding mass from LC-MS analysis of compound 11. Gradient: 5—95% CH_3CN/H_2O with 0.1% TFA over 15 min at a flow rate of 0.6 mL/min. ESI: calculated for $C_{43}H_{64}N_7O_{10}^+$ [M+H$^+$]: 838.5; found: 838.2

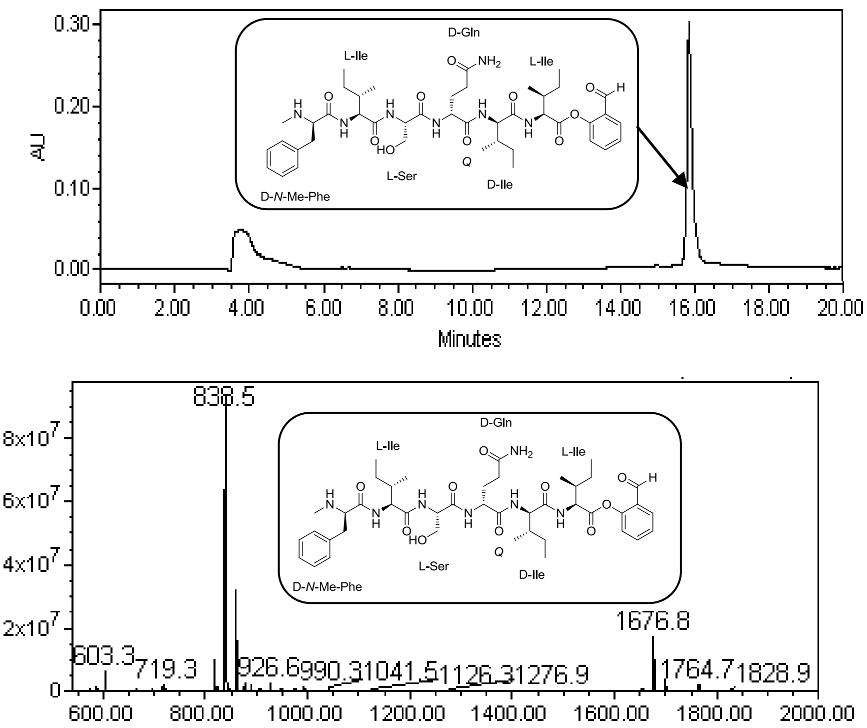

Supplementary Figure 34. UV trace and corresponding mass from LC-MS analysis of compound S7. Gradient: 5—95% CH_3CN/H_2O with 0.1% TFA over 15 min at a flow rate of 0.6 mL/min. ESI: calculated for $C_{43}H_{64}N_7O_{10}^+$ [M+H$^+$]: 838.5; found: 838.5

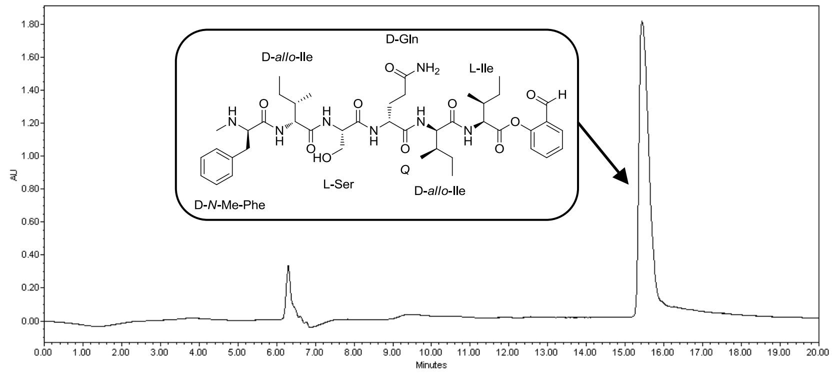

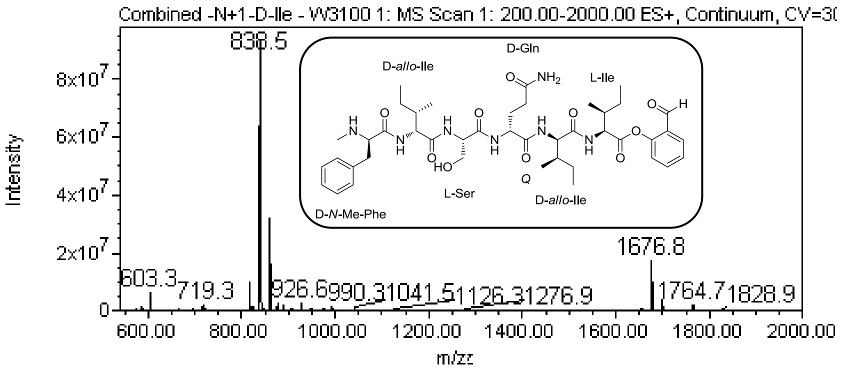

Supplementary Figure 35. UV trace and corresponding mass from LC-MS analysis of compound S8. Gradient: 5—95% CH_3CN/H_2O with 0.1% TFA over 15 min at a flow rate of 0.6 mL/min. ESI: calculated for $C_{43}H_{64}N_7O_{10}^+$ [M+H$^+$]: 838.5; found: 838.5

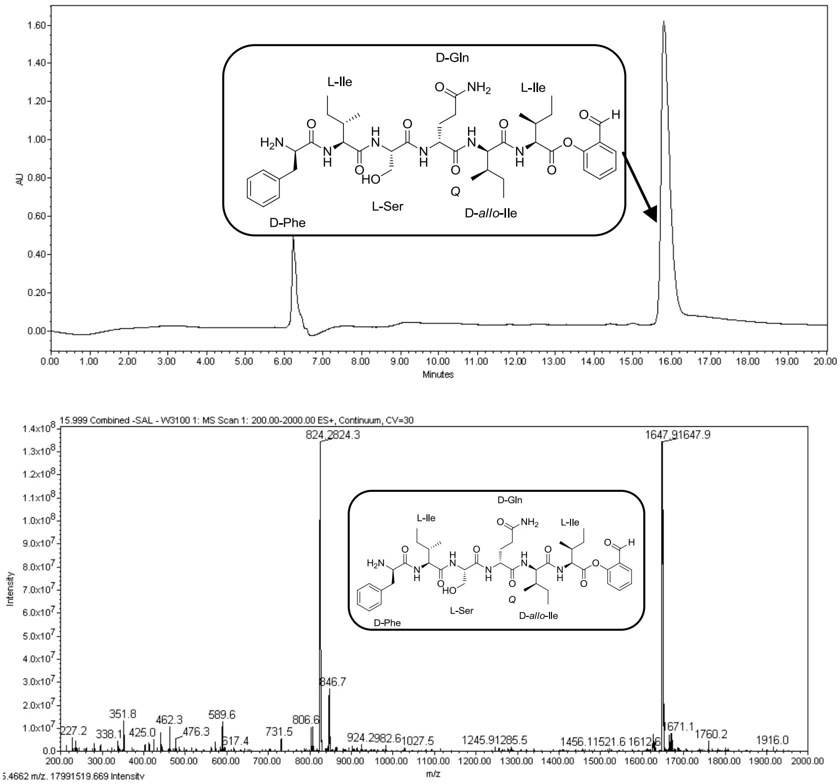

Supplementary Figure 36. UV trace and corresponding mass from LC-MS analysis of compound S9. Gradient: 5—95% CH_3CN/H_2O with 0.1% TFA over 15 min at a flow rate of 0.6 mL/min. ESI: calculated for $C_{42}H_{62}N_7O_{10}^+$ [M+H$^+$]: 824.5; found: 824.3

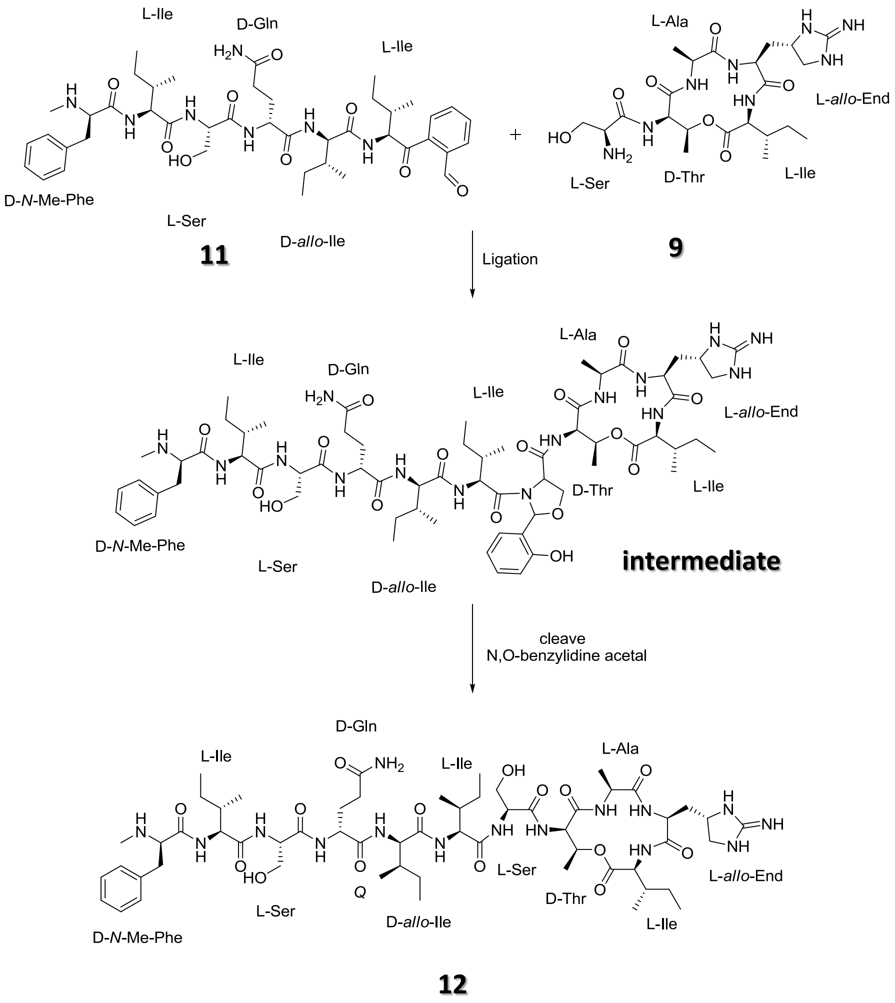

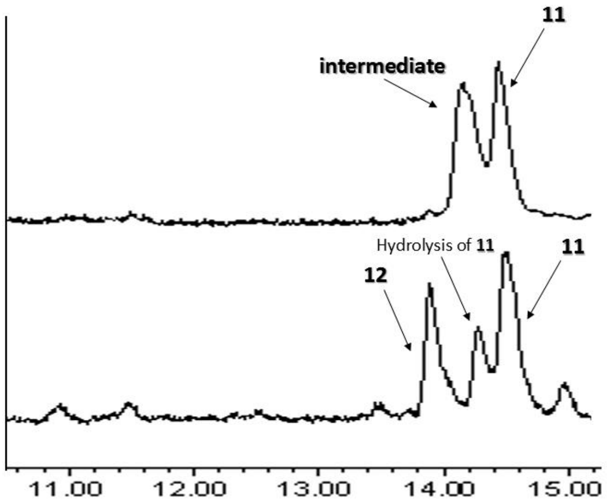

Supplementary Figure 37. UV trace from LC-MS analysis of Serine ligation and acidification. Gradient: 5—95% CH_3CN/H_2O with 0.1% TFA over 15 min at a flow rate of 0.6 mL/min.

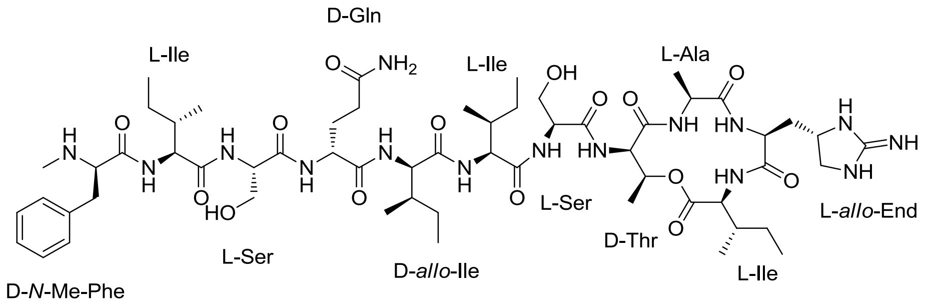

12

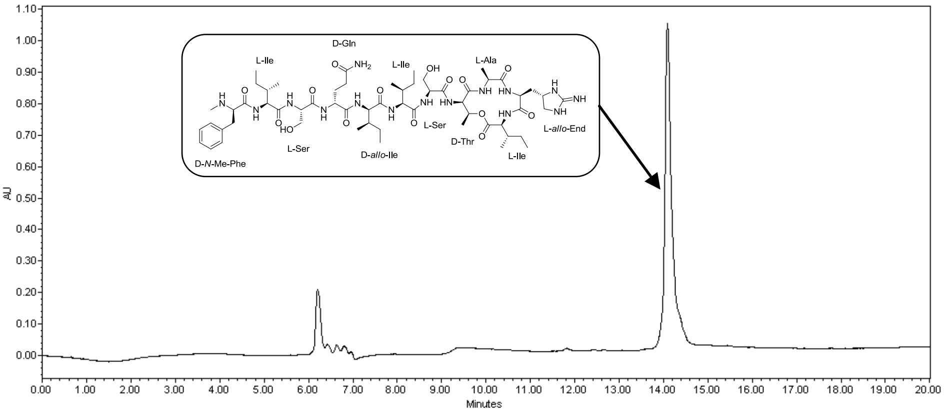

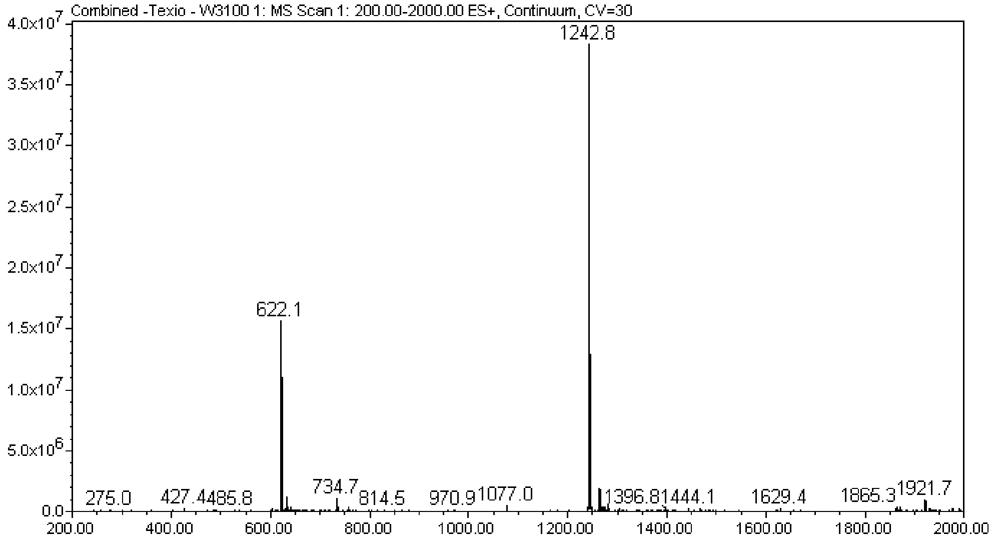

Supplementary Figure 38. UV trace and corresponding masses from LC-MS analysis of pure compound 12. Gradient: 5—95% CH_3CN/H_2O with 0.1% TFA over 15 min at a flow rate of 0.6 mL/min. ESI: calculated for $C_{58}H_{96}N_{15}O_{15}^{+}$ [M+H^{+}]: 1242.7; found: 1242.8

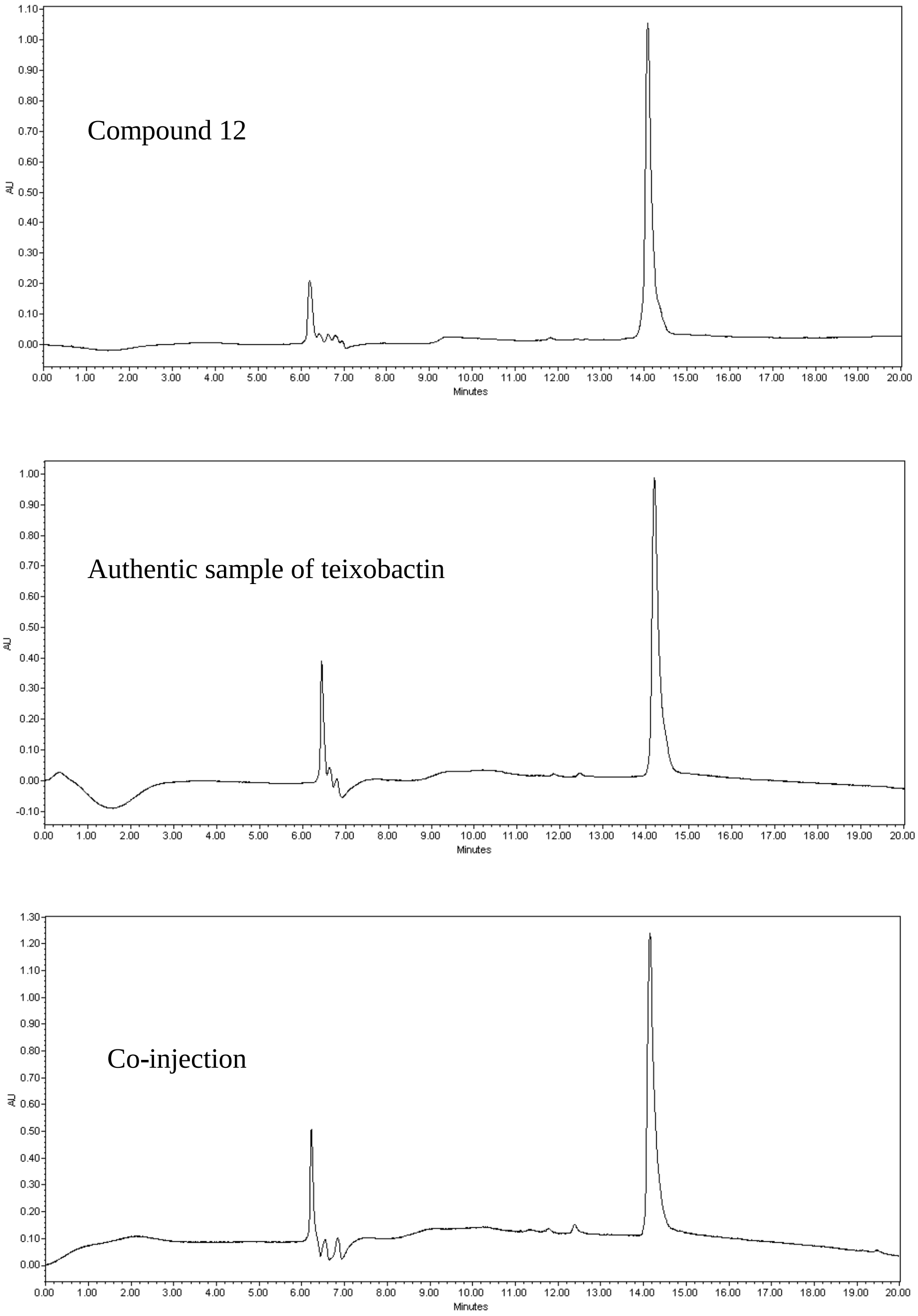

Supplementary Figure 39. UV traces from HPLC analysis of authentic sample of teixobactin and compound 12. Gradient: 5—95% CH_3CN/H_2O with 0.1% TFA over 15 min at a flow rate of 0.6 mL/min.

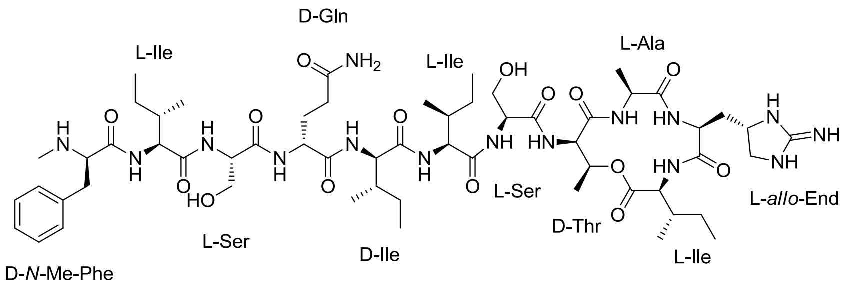

13

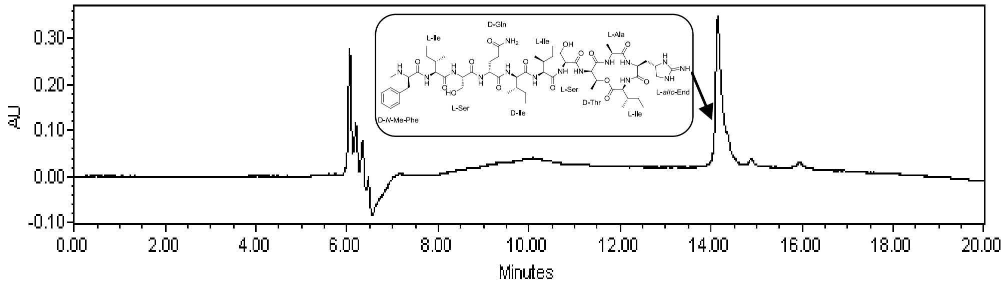

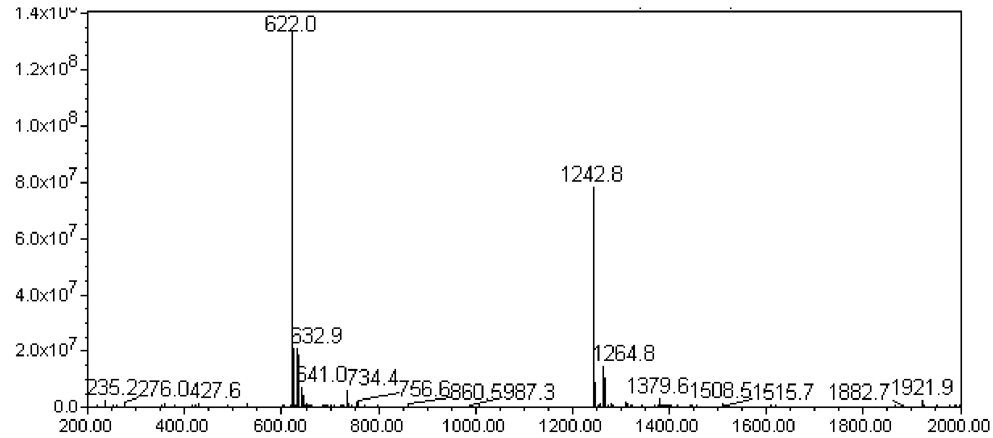

Supplementary Figure 40. UV trace and corresponding mass from LC-MS analysis of compound 13. Gradient: 5—95% CH_3CN/H_2O with 0.1% TFA over 15 min at a flow rate of 0.6 mL/min. ESI: calculated for $C_{58}H_{96}N_{15}O_{15}^+$ [M+H$^+$]: 1242.7; found: 1242.8

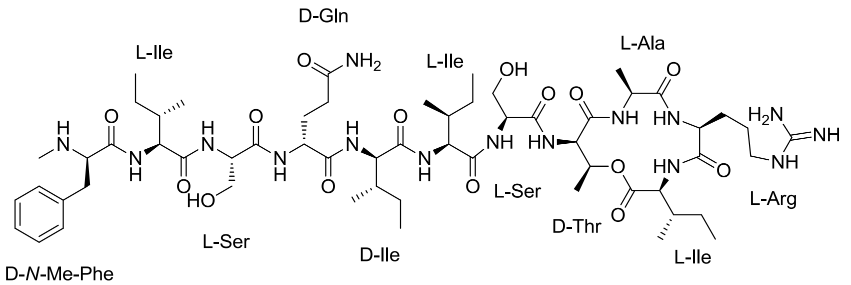

14

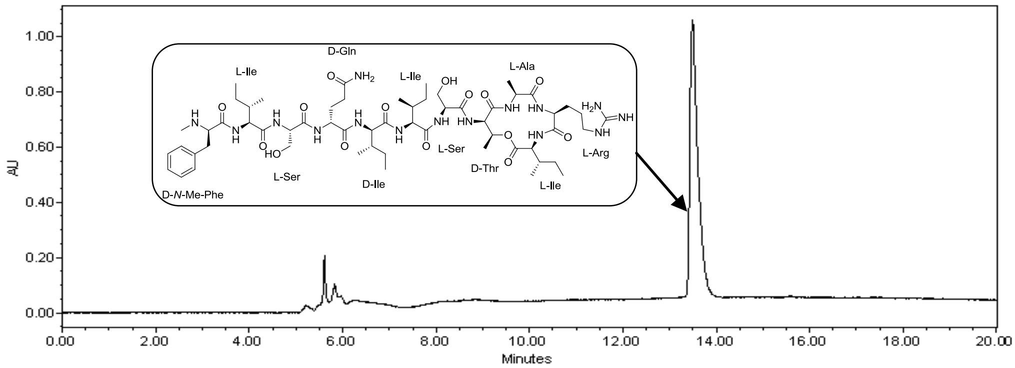

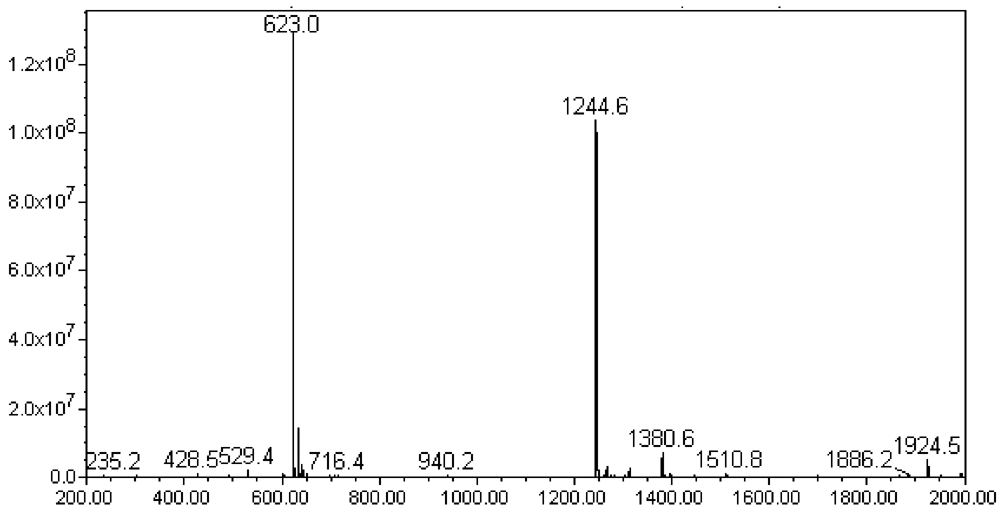

Supplementary Figure 41. UV trace and corresponding mass from LC-MS analysis of compound 14. Gradient: 5—95% CH_3CN/H_2O with 0.1% TFA over 15 min at a flow rate of 0.6 mL/min. ESI: calculated for $C_{58}H_{98}N_{15}O_{15}^+$ [M+H$^+$]: 1244.7; found: 1244.6

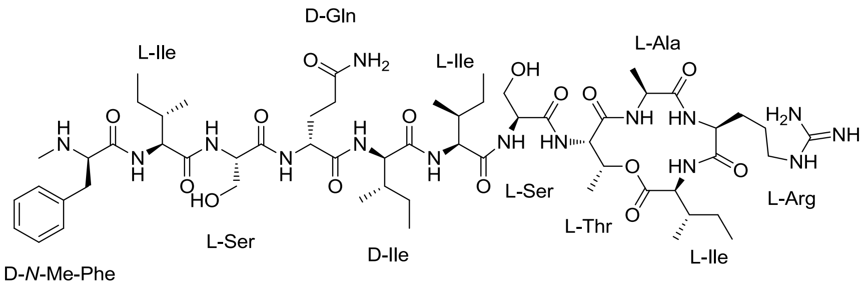

15

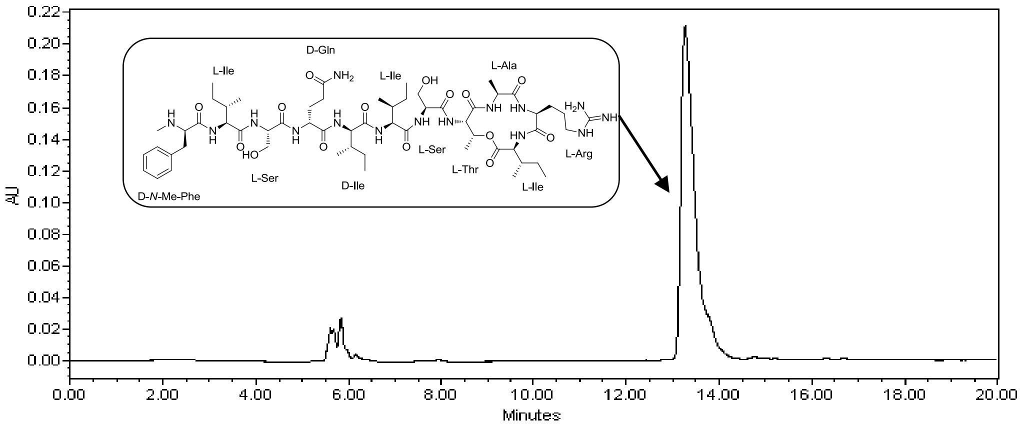

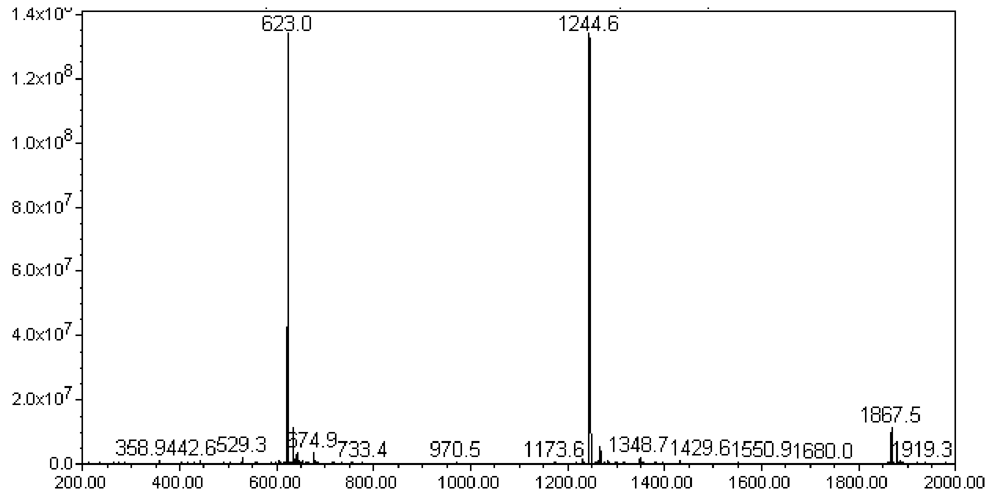

Supplementary Figure 42. UV trace and corresponding mass from LC-MS analysis of compound 15. Gradient: 5—95% CH_3CN/H_2O with 0.1% TFA over 15 min at a flow rate of 0.6 mL/min. ESI: calculated for $C_{58}H_{98}N_{15}O_{15}^+$ [M+H$^+$]: 1244.7; found: 1244.6

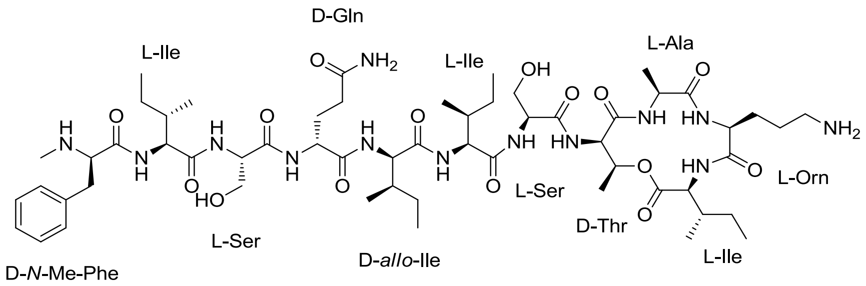

16

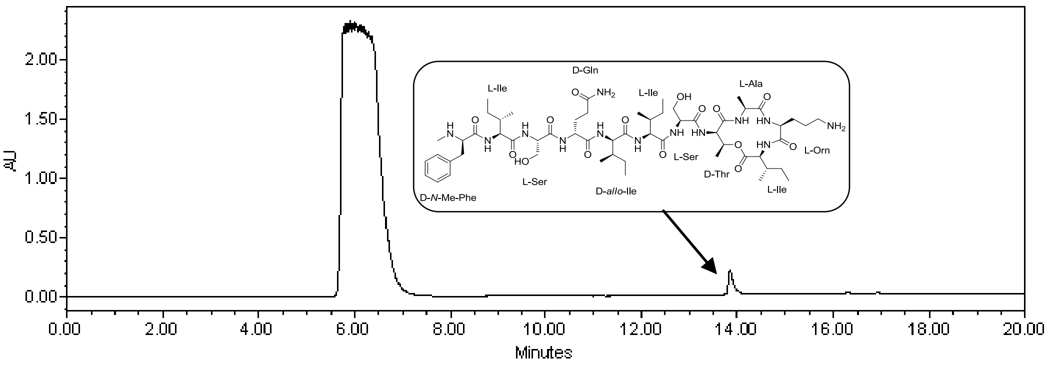

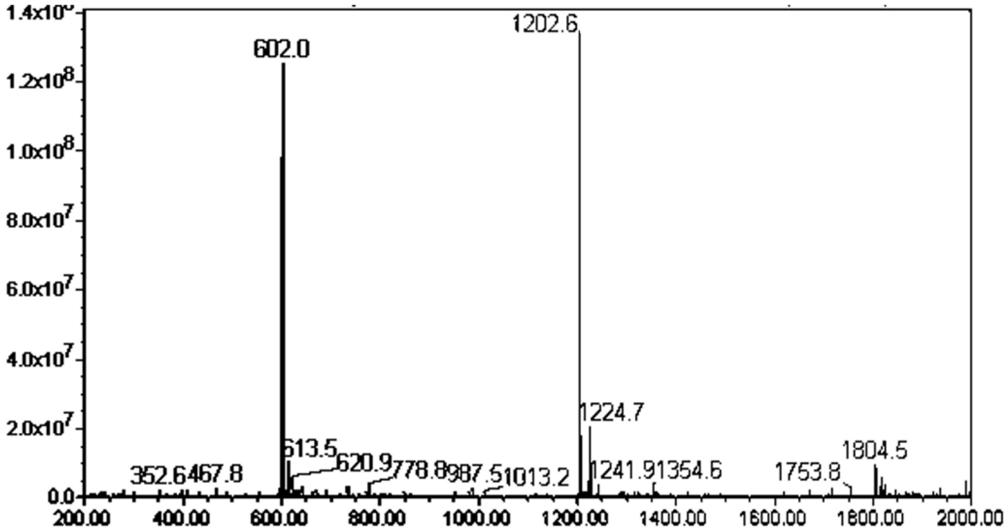

Supplementary Figure 43. UV trace and corresponding mass from LC-MS analysis of compound 16. Gradient: 5—95% CH_3CN/H_2O with 0.1% TFA over 15 min at a flow rate of 0.6 mL/min. ESI: calculated for $C_{57}H_{96}N_{13}O_{15}^{+}$ [M+H^{+}]: 1202.7; found: 1202.6

Supplementary Table 1. ^{1}H NMR signals of compound 12 (HCl Salt) and reported Teixobactin.[1]

^{1}H NMR signals (DMSO-d_6)			
Compound **12** [δ_H (mult., *J* in Hz)]	Reported[1] [δ_H (mult., *J* in Hz)]	Compound **12** [δ_H (mult., *J* in Hz)]	Reported[1] [δ_H (mult., *J* in Hz)]
2.5 (3H, t, 5.2)	2.5 (3H, br s)	7.8 (1H, d, 9.1)	7.8 (1H, d, 8.8)
4.2 (1H, br s)	4.2 (1H, dd, 9.4, 5.3)	1.8 (1H, m)	1.8 (1H, m)
3.0 (1H, dd, 13.08, 10.02)	3.0 (1H, dd, 13.2, 9.4)	0.8 (3H, m)	0.8 (3H, m)
3.1 (1H, dd, 13.2, 5.1)	3.1 (1H, dd 13.2, 5.3)	1.1 (1H, m)	1.1 (1H, m)
7.2 (2H, m)	7.2 (2H, m)	1.4 (1H, m)	1.4 (1H, m)
7.3 (2H, m)	7.3 (2H, m)	0.8 (3H, m)	0.9 (3H, m)
7.3 (1H, m)	7.3 (1H, m)	4.4 (1H, m)	4.5 (1H, dt, 5.0, 5.2)
4.1 (1H, dd, 7.70, 7.57)	4.1 (1H, dd, 7.8, 7.2)	8.7 (1H, d, 9.8) NH	8.4 (1H, d, 5.2) NH
8.8 (1H, d, 10.2) NH	8.4 (1H, d, 7.2) NH	3.6 (1H, m)	3.6 (1H, m)
1.5 (1H, m)	1.6 (1H, m)	3.8 (1H, m)	3.8 (1H, dd, 10.8, 5.0)
0.6 (1H, d, 6.76)	0.6 (1H, d, 6.7)	4.6 (1H, d, 9.3)	4.6 (1H, dd, 9.5, 2.2)
0.8 (1H, m)	0.8 (1H, m)	5.3 (1H, m)	5.4 (1H, dq, 2.2, 6.4)
1.1 (1H, m)	1.1 (1H, m)	1.1 (3H, d, 6.4)	1.1 (3H, d, 6.4)
0.6 (3H, t, 7.27)	0.7 (3H, t, 7.1)	3.9 (1H, m)	4.0 (1H, dq, 5.1, 7.5)
4.3 (1H, m)	4.3 (1H, m)	8.1 (1H, d, 5.1) NH	8.1 (1H, d, 5.1) NH
7.9 (1H, d, 8.02) NH	7.9 (1H, d, 7.9) NH	1.3 (3H, d, 7.3)	1.3 (3H, d, 7.5)
3.6 (1H, dd, 10.9, 5.6)	3.6 (1H, dd, 10.08, 5.6)	4.4 (1H, m)	4.4 (1H, m)
3.6 (1H, m)	3.6 (1H, dd, m)	8.6 (1H, d, 8.4) NH	8.3 (1H, d, 9.1) NH
4.3 (1H, m)	4.3 (1H, m)	2.0 (2H, m)	2.0 (2H, m)
7.9 (1H, d, 7.4) NH	7.9 (1H, d, 7.9) NH	3.9 (1H, m)	3.9 (1H, m)
2.2 (1H, m)	2.1 (2H, m)	9.0 (1H, br s) NH	8.0 (1H, br s) NH
1.7 (1H, m)	1.7 (1H, m)	3.4 (overlapped with water peak)	3.4 (1H, dd, 9.4, 7.7)
1.9 (1H, m)	1.9 (1H, m)	3.5 (1H, t, 5.5)	3.6 (1H, t, 9.4)

6.7 (1H, br s)	6.6 (1H, br s)	9.1 (1H, br s) NH	8.1, (1H, br s) NH
7.2 (1H, br s)	7.1 (1H, br s)	7.9 (2H, br s) NH	7.8 (2H, br s) NH
4.4 (1H, m)	4.4 (1H, m)	4.0 (1H, t, 9.8)	4.0 (1H, t, 9.4)
7.7 (1H, br s)	7.7 (1H, d, 8.8)	8.0 (1H, d, 7.8) NH	8.0 (1H, d, 9.4) NH
1.8 (2H, m)	1.8 (2H, m)	1.8 (1H, m)	1.8 (1H, m)
0.8 (3H, m)	0.8 (3H, m)	0.8 (3H, m) NH	0.8 (3H, m) NH
1.1 (1H, m)	1.1 (1H, m)	0.8 (1H, m)	0.8 (1H, m)
1.3 (1H, m)	1.3 (1H, m)	1.1 (1H, m)	1.1 (1H, m)
0.8 (3H, m)	0.8 (3H, m)	0.8 (3H, m)	0.8(3H, m)
4.3 (1H, m)	4.3 (1H, m)		

Supplementary Table 2. ^{13}C NMR signals of compound 12 (HCl Salt) and reported Teixobactin.[1]

^{13}C NMR signals (DMSO-d_6)			
Compound **12** δ_c	Reported[1] δ_c	Compound **12** δ_c	Reported[1] δ_c
31.9	31.9	57.3	57.3
61.8	61.9	37.2	36.9
36.4	36.4	15.6	15.4
135.1	135.0	25.5	25.3
129.8	129.7	11.4	11.2
129.0	128.9	171.6	171.6
127.7	127.5	56.2	56.5
167.1	167.1	64.3	62.7
57.9	57.9	171.6	171.7
36.7	36.5	56.1	56.2
15.6	15.5	70.8	71.2
24.3	24.4	15.9	15.9
11.7	11.3	168.5	168.9
170.7	170.6	52.3	52.2
55.6	55.6	17.3	17.1
62.4	62.4	173.1	173.1
170.2	170.2	52.3	52.2
52.5	52.7	37.5	37.2
31.9	31.9	53.9	53.5
28.7	28.4	48.5	48.3
174.5	174.4	159.5	160.0
171.1	170.9	171.9	171.8
55.9	56.8	57.8	57.8
37.6	37.4	35.9	36.3
14.7	14.7	16.0	16.0
26.4	26.2	24.8	24.5
10.6	10.6	12.0	11.8
171.3	171.4	169.8	169.3

Supplementary Methods

List of abbreviations

AcOH	Acetic acid
Alloc-Cl	Allyl chloroformate
CH_3I	Methyl iodide
DCM	Dichloromethane
DIC	*N,N'*-Diisopropylcarbodiimide
DIEA	Diisopropylethylamine
DMAP	4-Dimethylaminopyridine
DMF	*N,N'*-Dimethylformamide
EDCI	1-Ethyl-3-(3-dimethylaminopropyl)carbodiimide
ESI	Electrospray ionization
EtOAc	Ethyl acetate
Fmoc-OSu	N-(9-Fluorenylmethoxycarbonyloxy)succinimide
HATU	O-(7-Azabenzotriazole-1-yl)-1,1,3,3-tetramethyluronium hexafluorophosphate
HCl	Hydrochloric acid
HCOOH	Formic acid
HOAT	1-Hydroxy-7-azabenzotriazole
HOBT	1-Hydroxybenzotriazole
HPLC	High-performance liquid chromatography
HRMS	High-resolution mass spectrometry
LC-MS	High performance liquid chromatography mass spectrometry
MeOH	Methanol
Me_2S	Dimethyl sulfide
NaH	Sodium hydride
NMR	Nuclear magnetic resonance
OxymaPure	Ethyl (hydroxyimino)cyanoacetate
$Pd(PPh_3)_4$	Tetrakis(triphenylphosphine)palladium(0)
PMB-Cl	4-Methoxybenzoyl chloride
TFA	Trifluoroacetic acid
TFE	2,2,2-Trifluoroethanol
THF	Tetrahydrofuran
TIPS	Triisopropylsilane
TMSOTf	Trimethylsilyl trifluoromethanesulfonate

Materials and analytical methods

All natural amino acids and coupling reagents (Aldrich and GL Biochem) were commercially available and used without further purification. Compound **S1**[2] (Boc-L-*allo*-End(Cbz)$_2$-OH) was prepared by Yu Yuan's group in University of Central Florida. The authentic sample of teixobactin was supplied by Prof. Kim Lewis from Northeastern University. All solvents were HPLC grade (DUKSAN) or analytical reagent grade (RCI). Anhydrous dichloromethane (DCM) was distilled in the presence of calcium hydride (CaH_2).

Analytical HPLC was performed on a Waters system, using a Vydac 218TP™ C18 column (5 µm, 4.6×250 mm). Preparative HPLC was performed on a Waters system, using a Vydac 218TP™ C18 column (10 µm, 10×250 mm) or a Vydac 218TP™ C18 column (10 µm, 22×250 mm). Buffer A: 0.1% TFA in acetonitrile; buffer B: 0.1% TFA in H_2O. ^{1}H and ^{13}C experiments were performed on Bruker Avance DRX 400 FT-NMR instrument at 400MHz for ^{1}H NMR and 100MHz for ^{13}C NMR; or Bruker Avance DRX 500 FT-NMR instrument at 500MHz for ^{1}H NMR and 125MHz for ^{13}C NMR; or Bruker Avance 600 FT-NMR spectrometer at 600 MHz for ^{1}H NMR and 150 MHz for ^{13}C NMR.

Experimental procedures

1. **Synthesis of Building Blocks**

Synthesis of Fmoc-Ile-$_D$Thr(NH-Alloc)-OH (4a)

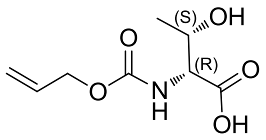

1a

To an ice-water cooled solution of D-threonine (1.0 g, 9.9 mmol) in 15 mL THF, Na_2CO_3 (5.25 g, 49.5 mmol) in 15 mL H_2O was added slowly. 10 minutes later, Alloc-Cl (1.2 mL, 19.8 mmol) was added and the reaction was allowed to warm up and stirred for 12 hours at room temperature. The reaction mixture was concentrated under v*acuo*. The solution was adjusted to PH=3 with 1.0 M HCl in water and then extracted with ethyl acetate (100 mL×5). The organic phase was dried with anhydrous Na_2SO_4 and concentrated under low pressure. The crude residue was purified by flash column chromatography on silica gel (hexanes: EtOAc=1:1, with 0.5% AcOH) to produce compound **1a** (1.7 g, 86% yield). ^{1}H NMR (400 MHz, $CDCl_3$) δ 7.92 (1H, s), 6.17 (1H , d, *J* = 9.1 Hz), δ 5.85 (1H, ddd, *J* = 22.5, 10.8, 5.5 Hz), 5.26 (1H, dd, *J* = 17.2, 0.9 Hz), 5.16 (1H , d, *J* = 10.4 Hz), 4.53 (2H , d, *J* = 5.0 Hz), 4.37 (1H, d, *J* = 6.0 Hz), 4.28 (1H, d, *J* = 9.4 Hz), 1.18 (3H, d, *J* = 6.2 Hz). ^{13}C NMR (101 MHz, $CDCl_3$) δ 174.31, 157.21, 132.34, 117.92, 67.97, 66.23, 59.08, 19.29. HRMS (ESI): calculated for $C_8H_{13}NaNO_5^+$ [M+Na$^+$]: 226.0691; found: 226.0688.

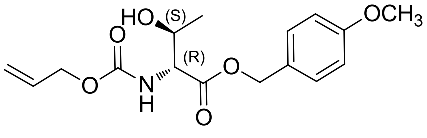

2a

Compound **1a** (1.5 g, 7.4 mmol) was dissolved in 5 mL DMF, then para-methoxybenzoyl chloride (PMB-Cl, 1.2 mL, 8.9 mmol) and $KHCO_3$ (1.5 g, 14.8 mmol) was added to the solution. The reaction mixture was stirred at 45 ^{o}C for 36 hours. The reaction solution was diluted with EtOAc (300 mL), and washed with saturated NH_4Cl aqueous solution (50 mL ×5). The organic phase was dried with anhydrous Na_2SO_4 and concentrated under low pressure. The crude residue was purified by flash column chromatography on silica gel (hexanes: EtOAc=4:1) to afford compound **2a** (2.1 g, 87% yield). ^{1}H NMR (400 MHz, $CDCl_3$) δ 7.29 (2H, d, *J* = 8.6 Hz), 6.90 –

6.87 (2H, m), 5.91 (1H, ddd, *J* = 22.2, 10.6, 5.4 Hz), 5.59 (1H, d, *J* = 8.7 Hz), 5.31 (1H, d, *J* = 17.1 Hz), 5.21 (1H, d, *J* = 10.4 Hz), 5.14 (2H, d, *J* = 6.0 Hz), 4.58 (2H, d, *J* = 5.5 Hz), 4.33 (2H, d, *J*= 7.4 Hz), 3.81 (3H, s), 1.22 (3H, d, *J* = 6.4 Hz).^{13}C NMR (101 MHz, $CDCl_3$) δ 171.08, 159.81, 132.53, 130.15, 127.32, 117.85, 114.02, 68.07, 67.26, 66.02, 59.16, 55.29, 19.89. HRMS (ESI): calculated for $C_{16}H_{21}NaNO_6^+$ [M+Na$^+$]: 346.1267; found: 346.1260.

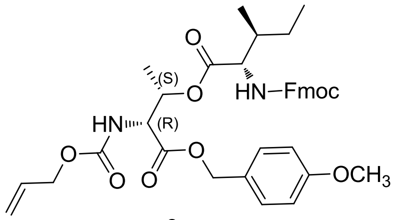

3a

Compound **2a** (2.0 g, 6.2 mmol) was dissolved in 20 mL anhydrous DCM. Then the mixture of Fmoc-Ile-OH (8.8 g, 24.8 mmol), EDCI(3.8 g, 24.8 mmol) and DMAP (75 mg, 0.6 mmol) in 100 mL anhydrous DCM was added to the solution and stirred at room temperature for 6 hours. The reaction mixture was washed by 1.0 M HCl (20 mL ×3) and brine (20 mL×3). The organic phase was dried with anhydrous Na_2SO_4 and concentrated under low pressure. The crude residue was purified by flash column chromatography on silica gel (hexanes: EtOAc=4:1) to afford compound **3a** (3.2 g, 79% yield). ^{1}H NMR (400 MHz, $CDCl_3$) δ 7.76 (2H, d, *J* = 7.6 Hz), 7.60 (2H, dd, *J* = 7.0, 3.9 Hz), 7.39 (2H, t, *J* = 7.4 Hz), 7.31 (2H, t, *J* = 7.4 Hz), 7.26 (2H, d, *J* = 8.0 Hz), 6.87 (2H, d, *J* = 8.5 Hz), 5.90 (1H, ddt, *J* = 16.2, 10.8, 5.6 Hz), 5.47 (2H, d, *J* = 8.9 Hz), 5.31 (1H, d, *J* = 17.2 Hz), 5.22 (2H, t, *J*= 8.2 Hz), 5.06 (2H, dd, *J* = 38.9, 11.8 Hz), 4.58 (2H, d, *J* = 5.5 Hz), 4.51 (1H, dd, *J* = 9.5, 1.8 Hz), 4.45 – 4.36 (2H, m), 4.24 (2H, dt, *J* = 10.3, 5.9 Hz), 3.79 (3H, s) 1.82 (1H, m), 1.31 (4H, d, *J* = 6.4 Hz), 1.11 (1H ,m), 0.91 (6H, dd, *J* = 10.8, 7.1 Hz).^{13}C NMR (101 MHz, $CDCl_3$) δ 170.79, 169.58, 159.93, 156.38, 156.19, 143.94, 143.78, 141.33, 132.39, 130.29, 127.73, 127.09, 126.88, 125.10, 120.01, 118.13, 114.07, 71.91, 67.69, 67.08, 66.23, 58.73, 57.55, 55.30, 47.21, 37.56, 24.66, 17.02, 15.47, 11.55. HRMS (ESI): calculated for $C_{37}H_{42}NaN_2O_9^+$ [M+Na$^+$]: 681.2788; found: 681.2777.

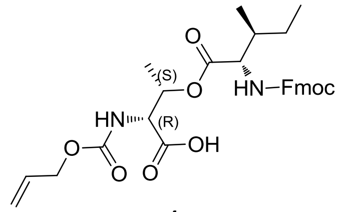

4a

Compound **3a** (3.0 g, 4.6 mmol) was dissolved in 5 mL TFA. The reaction solution was stirred for 3 min to remove the PMB group. TFA was blown away by a condensed air stream and evaporated several times with benzene. The crude residue was dissolved in 5 mL DCM and purified by flash column chromatography on silica gel (hexanes: EtOAc=2:1, with 0.5% AcOH) to produce compound **4a** (2.4 g, 98% yield). ^{1}H NMR (400 MHz, MeOD) δ 7.60 (2H, d, *J* = 7.4 Hz), 7.55 – 7.45 (2H, m), 7.21 (2H, dd, *J* = 13.6, 6.3 Hz), 7.14 (2H, t, *J* = 7.4 Hz), 5.79 (1H, ddd, *J* = 22.2, 10.6, 5.3 Hz), 5.35 (1H, dd, *J* = 6.2, 2.6 Hz), 5.18 (1H, d, *J* = 17.2 Hz), 5.04 (1H, d, *J* = 10.3 Hz), 4.87 (2H, s), 4.43 (2H, d, *J* = 5.1 Hz), 4.32 (1H, dd, *J* = 7.3, 4.4 Hz), 4.26 – 4.17 (2H, m), 4.08 – 3.95 (2H, m), 1.72 (1H, dd, *J* = 6.0, 3.2 Hz), 1.36 – 1.24 (1H, m), 1.18 (3H, d, *J* = 6.3 Hz),

1.04 (1H, dd, J = 13.3, 8.3 Hz), 0.77 (6H, t, J = 6.5 Hz).^{13}C NMR (101 MHz, MeOD) δ 172.38, 172.18, 158.68, 158.46, 145.07, 144.78, 142.25, 133.86, 128.49, 127.90, 127.88, 125.96, 120.65, 117.55, 72.56, 67.72, 66.59, 60.15, 58.66, 37.86, 25.63, 16.91, 15.68, 11.42. HRMS (ESI): calculated for $C_{29}H_{34}NaN_2O_8^+$ [M+Na$^+$]: 561.2213; found: 561.2210.

Synthesis of Fmoc-Ile-*allo*-$_L$Thr(NH-Alloc)-OH (4b) and Fmoc-Ile-$_L$Thr(NH-Alloc)-OH (4c)

Fmoc-Ile- *allo*-$_L$Thr(NH-Alloc)-OH (**4b**) and Fmoc-Ile-$_L$Thr(NH-Alloc)-OH (**4c**) and were synthesized with the same synthetic strategy as Fmoc-Ile-$_D$Thr(NH-Alloc)-OH (**4a**).

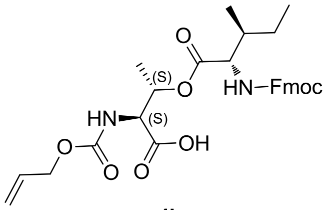

4b

Fmoc-Ile-*allo*-$_L$Thr(NH-Alloc)-OH (**4b**): Flash column chromatography on silica gel (hexanes: EtOAc=2:1, with 0.5% AcOH) as white solid (2.2 g, 56% yield over four steps). ^{1}H NMR (400 MHz, MeOD) δ 7.79 (2H, d, J = 7.5 Hz), 7.70 (2H, t, J = 6.6 Hz), 7.38 (2H, t, J = 7.4 Hz), 7.30 (2H, t, J = 7.4 Hz), 5.90 (1H, ddt, J = 16.0, 10.6, 5.3 Hz), 5.35 (1H, s), 5.27 (1H, d, J = 17.2 Hz), 5.14 (1H, d, J = 10.5 Hz), 4.52 (2H, s), 4.36 (3H, dd, J = 16.3, 8.7 Hz), 4.22 (1H, t, J = 7.0 Hz), 4.16 – 4.08 (1H, m), 1.89 (1H, s), 1.53 – 1.42 (1H, m), 1.27 (3H, d, J = 3.8 Hz,), 1.20 (1H, d, J = 8.6 Hz), 0.93 (6H, dd, J = 7.0, 3.2 Hz).^{13}C NMR (101 MHz, MeOD) δ 172.27, 158.51, 157.91, 154.14, 145.22, 144.95, 142.37, 134.19, 128.55, 127.97, 127.93, 126.13, 120.67, 117.38, 73.42, 67.84, 66.33, 60.47, 59.80, 38.06, 30.53, 25.75, 15.77, 15.33, 11.55.HRMS (ESI): calculated for $C_{29}H_{34}NaN_2O_8^+$ [M+Na$^+$]: 561.2213; found: 561.2210.

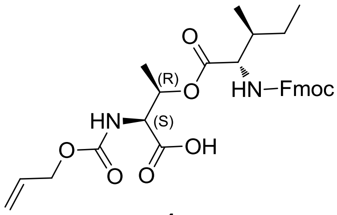

4c

Fmoc-Ile-$_L$Thr(NH-Alloc)-OH (**4c**): Flash column chromatography on silica gel (hexanes: EtOAc=2:1, with 0.5% AcOH) as white solid (2.4 g, 57% yield over four steps). ^{1}H NMR (400 MHz, MeOD) δ 7.62 (2H, d, J = 7.5 Hz), 7.50 (2H, t, J = 7.7 Hz), 7.23 (2H, t, J = 7.3 Hz), 7.18 – 7.13 (2H, m), 5.81 (1H, ddd, J = 22.5, 10.7, 5.4 Hz), 5.37 (1H, dd, J = 6.3, 2.8 Hz), 5.20 (1H, dd, J = 17.2, 1.3 Hz), 5.06 (1H, dd, J = 10.5, 1.1 Hz), 4.45 (2H, d, J = 5.4 Hz), 4.37 – 4.30 (1H, m), 4.25 (2H, d, J = 6.8 Hz), 4.06 (1H, t, J = 6.7 Hz), 3.98 (1H, dd, J = 11.2, 5.0 Hz), 1.71 – 1.61 (1H, m), 1.33 (1H, ddd, J = 13.3, 7.4, 4.0 Hz), 1.14 (3H, d, J = 6.4 Hz), 1.03 (1H, td, J = 15.9, 7.4 Hz), 0.77 (6H, dd, J = 6.8, 5.6 Hz).^{13}C NMR (101 MHz, MeOD) δ 172.52, 171.83, 171.43, 158.70, 158.24, 158.19, 145.06, 144.78, 142.32, 142.29, 133.88, 128.51, 127.89, 125.91, 125.85, 120.69, 117.64, 72.23, 67.53, 66.65, 60.39, 60.30, 58.70, 58.61, 38.05, 25.82, 17.01, 15.65, 11.24. HRMS

(ESI): calculated for $C_{29}H_{34}NaN_2O_8^+$ [M+Na$^+$]: 561.2213; found: 561.2210.

Synthesis of Fmoc-L-*allo*-End(Cbz)$_2$-OH (S2)

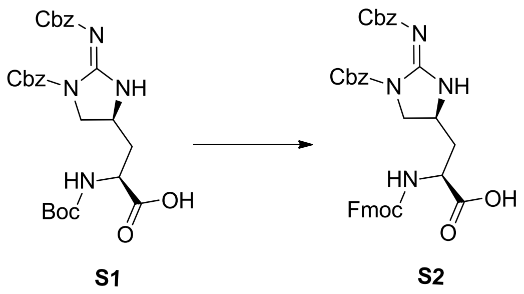

Compound **S1**[2] (200 mg, 0.37 mmol) was dissolved in1 mL TFA/DCM (*v/v*=9:1) solution and stirred for 1 hour. TFA/DCM was blown away by a condensed air stream. The reaction residue was dissolved in H_2O. Na_2CO_3 was added to the mixture and stirred for 5 min at 0 ^oC. Then a solution of Fmoc-OSu (189 mg, 0.56 mmol) in 2 mL dioxane was added into the reaction mixture which was allowed to warm up and stirred at room temperature for 18 hours. . The reaction mixture was concentrated under v*acuo*. The solution was adjusted to PH=2 with 1.0 M HCl in water and then extracted with ethyl acetate (25 mL ×5). The organic phase was dried with anhydrous $NaSO_4$ and concentrated under low pressure. The crude residue was purified by flash column chromatography on silica gel (DCM: MeOH=20:1, with 0.5% AcOH) to afford compound **S2** (205 mg, 84% yield). ^{1}H NMR (400 MHz, $CDCl_3$ with 1%MeOD) δ 7.68 (2H, d, *J* = 4.2 Hz), 7.54 (2H, d, *J* = 7.3 Hz), 7.30 (7H, m), 7.25 – 7.15 (7H, m), 5.13 (2H, d, *J* = 7.5 Hz), 5.04 (2H, s), 4.32 (2H, dt, *J* = 17.1, 10.4 Hz), 4.12 (2H, t, *J* = 6.6 Hz), 3.89 (1H, s), 3.41 – 3.31 (2H, m), 1.85 (2H, m).^{13}C NMR (101 MHz, $CDCl_3$ with 1%MeOD) δ 156.54, 151.50, 143.92, 143.71, 141.25, 135.93, 134.83, 128.65, 128.38, 128.23, 128.09, 127.66, 127.63, 127.04, 127.00, 125.19, 125.06, 119.92, 119.86, 68.46, 67.58, 66.76, 47.20, 39.85, 29.66. LRMS (ESI): calculated for $C_{37}H_{35}N_4O_8^+$ [M+H$^+$]: 663.24; found: 663.25. HRMS (ESI): calculated for $C_{29}H_{29}N_4O_6^+$ [M-Cbz]$^+$: 529.2009; found: 529.2152.

Synthesis of Boc-D-*N*-Me-Phe-OH (S3)[3]

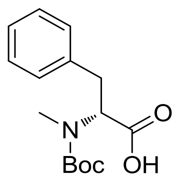

S3

To a solution of Boc-D-Phe-OH (1.0 g, 3.8 mmol) in 20mL dry THF, was added NaH (0.5 g, 20.8 mmol) and CH_3I (5.4 g, 2.4 mL, 38.0 mmol). The reaction mixture was stirred at room temperature for 24 hours. The reaction mixture was diluted with 200 mL H_2O and washed with ethyl acetate (50 mL ×3). The aqueous layer was acidified with 20% aqueous citric acid to PH=2 and extracted with ethyl acetate (50 mL ×3). The organic phase was concentrated under low pressure and purified by flash column chromatography on silica gel (hexanes: EtOAc=1:1, with 0.5% AcOH) to get compound **S3** (0.9 g, 85% yield). ^{1}H NMR (400 MHz, $CDCl_3$, two rotamers,

M/m=1.3:1) δ 8.78 (1H, s), 7.32 – 7.13 (5H, m), 4.96 (1H, dd, J = 11.1, 4.8 Hz), 4.62 (1H, dd, J = 11.0, 3.9 Hz), 3.32 (1H, ddd, J = 20.9, 14.4, 4.4 Hz), 3.03 (1H, dd, J = 26.0, 14.6 Hz), 2.75 (2H, s), 2.69 (1H, s), 1.36 (4H, s), 1.31 (5H, s). ^{13}C NMR (101 MHz, $CDCl_3$, two rotamers, M/m=1.3:1) δ 175.09, 174.66, 156.09, 155.24, 137.44, 137.09, 136.63, 128.85, 128.74, 128.41, 128.24, 126.52, 126.41, 80.72, 80.34, 61.36, 59.61, 35.07, 34.59, 32.31, 32.08, 28.06, 27.93.[3]

2. Synthesis of Teixobactin and its analogs

Solid-phase peptide synthesis of linear pentapeptide 8

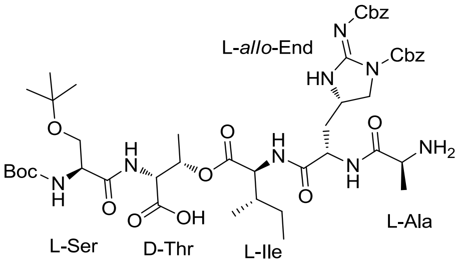

8

100 mg 2-chlorotrityl resin (0.5 mmol/g) was placed in a 6 mL polypropylene syringe with a polyethylene filter in the bottom. It was swollen with DCM for 1 hour. Then it was washed by DMF (3 × 2 mL) and DCM (3 × 2 mL). The first building block was added by using Fmoc-Ile-$_{D}$Thr(NH-Alloc)-OH (53.8 mg, 0.1 mmol) and DIEA (34.8 μL, 0.2 mmol) in 2 mL DCM and shook for 1 hour. Then 80 μL MeOH was added and shook for another 20 min. Then the resin was washed by DCM (3 × 2 mL) and DMF (3 × 2 mL).

The alloc deprotection was achieved by using $Pd(PPh_3)_4$ (11.6 mg, 0.01 mmol) and phenylsilane (154 μL, 1.25 mmol) in 2 mL anhydrous DCM and shook for 1 hour under argon environment. The following amino acid was coupled by using Boc-Ser(OtBu)-OH (52.2 mg, 0.2 mmol, 4 equiv), HATU (76.0 mg, 0.2 mmol, 4 equiv), DIEA (34.8 μL, 0.4 mmol, 8 equiv) in 2 mL DMF and shook for 1 hour.

Then Fmoc was removed by shaken in 2 mL DMF (containing 20% piperidine) for 20 min. The resin was washed by DCM (3 × 2 mL, 1 min) and DMF (3 × 2 mL, 1 min). After a mini-cleavage by TFA/H_2O/TIPS (*v/v/v*=94:5:1) for 1 hour, the reaction was monitored by LC-MS. ESI: calculated for $C_{13}H_{26}N_3O_6^+$ [M+H$^+$]: 320.2; found: 320.2 (**Supplementary Figure 30**).

To the above resin, a solution of Fmoc-L-*allo*-End(Cbz)$_2$-OH (49.7 mg, 0.075 mmol, 1.5 equiv) in 500 μL DMF was added and shook up. Then a mixture of DIC (23.2 μL, 0.15 mmol, 3.0 equiv) and HOBT (23.0 mg, 0.15 mmol, 3.0 equiv) in 500 μL DMF was added and shaken for 10 hours. This coupling procedure was repeated two more times to confirm the reaction was completed. After a mini-cleavage by TFA/H_2O/TIPS (*v/v/v*=94:5:1) for 1 hour, the reaction was monitored by LC-MS. ESI: calculated for $C_{50}H_{58}N_7O_{13}^+$ [M+H$^+$]: 964.4; found: 964.4 (**Supplementary Figure 30**).

After Fmoc was removed in 2 mL DMF (containing 20% piperidine) for 10 min, the next amino acid was coupled using Fmoc-Ala-OH (62.2 mg, 0.2 mmol, 4 equiv), HATU (76.0 mg, 0.2 mmol, 4 equiv), DIEA (34.8 μL, 0.4 mmol, 8 equiv) in 2 mL DMF. The mixture was shaken for 1 hour and the resin was washed by DCM (3 × 2 mL, 1 min) and DMF (3 × 2 mL, 1 min). Then Fmoc was deprotected with 20% piperidine in DMF, and the resin was treated by a mixture of 3 mL DCM/AcOH/TFE (*v/v/v*=8:1:1) for 1.5 hours to obtain the crude linear pentapeptide **8** as white

solid (35 mg, 0.036 mmol).

Cyclization and removal of protecting groups

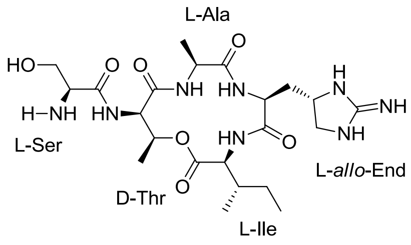

9

The crude protected peptide **8** (30 mg, 0.036 mmol) was dissolved in 360 mL anhydrous DCM at a concentration of 0.1 mmol/L. A mixture of DIEA (74.9 µL, 0.43 mmol, 12 equiv), HOAT (29.9 mg, 0.22 mmol, 6 equiv) and OxymaPure (31.3 mg, 0.22 mmol, 6 equiv) in 10 mL anhydrous DCM was added into reaction solution and stirred at 0 °C for 10 min. Then HATU (136.9 mg, 0.36 mmol, 10 equiv) was added.The reaction mixture was allowed to warm up slowly and stirred at room temperature for 24 hours.

DCM was evaporated under low pressure and the residue was treated with a mixture of 5 mL TFA/phenol/H_2O (*v/v/v*=95:2.5:2.5) for 1.5 hours. The mixture of TFA/phenol/H_2O was blown away by a condensed air stream and residue was washed by ethyl ether (20 mL × 3). The crude compound was dissolved in 1 mL MeOH/HCOOH(*v/v*=9:1) and hydrogenized by $Pd(OH)_2$ (90 mg, 10% on carbon) under H_2 (50 atm) for 10 hours. The reaction mixture was filtrated and concentrated in *vacuo*. The residue was purified by preparative HPLC (5-50% CH_3CN/H_2O over 30 min) to afford compound **9** (3.0 mg, 0.006 mmol, 18% yield based on the crude linear peptide **8**). LC-MS (ESI): calculated for $C_{22}H_{39}N_8O_7^+$ [M+H$^+$]: 527.3; found: 527.3 (**Supplementary Figure 31**).

Synthesis of Orn-containing cycle-tetrapeptide S4 and S5

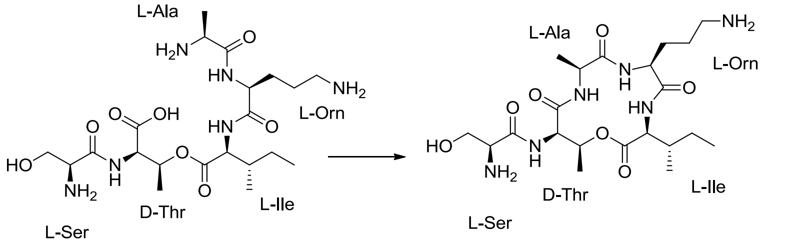

S4

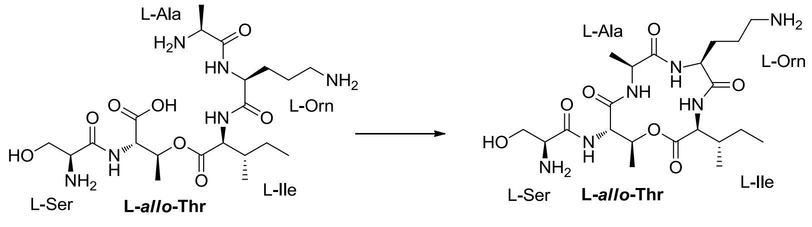

S5

Compounds **S4 and S5** were synthesized through the same method as compound **9**. Compound **S4** was monitored by LC-MS (ESI): calculated for $C_{21}H_{39}N_6O_7^+$ [M+H$^+$]: 487.3; found:

487.3 (**Supplementary Figure 32**). Compound **S5** was analyzed by LC-MS using the crude reaction mixture directly without purification. LC-MS (ESI): calculated for $C_{21}H_{39}N_6O_7^+$ [M+H$^+$]: 487.3; found: 487.4 (**Supplementary Figure 32**).

Synthesis of linear hexapeptide salicylaldehyde (SAL) esters 11 (D-*N*-Me-Phe-L-Ile-L-Ser-D-Gln-D-*allo*-Ile-L-Ile-SAL ester):[4]

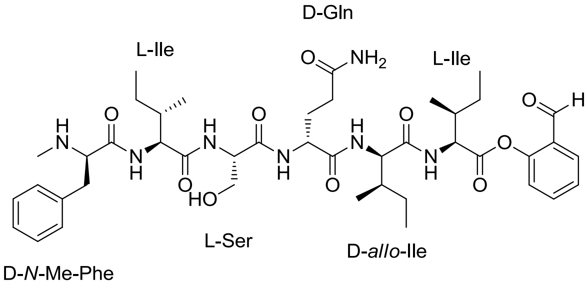

11

Aminomehtyl resin (Chemimpex, loading 1.1 mmol/g, 500 mg) was swollen in anhydrous DCM for 20 min. After DCM was drained, a mixture of compound 3-(2-acetoxyphenyl)acrylic acid (123.7 mg, 0.6 mmol, 2.0 equiv), HATU (228.1 mg, 0.6 mmol, 2.0 equiv) and DIEA (155.1 mg, 209.0 μL, 1.2 mmol, 4.0 equiv) in DMF was added. The reaction mixture was shaken for 12 hours at room temperature. The resin was washed with DMF (5 mL × 3) and DCM (5 mL × 3). Then acetic anhydride (56.2 mg, 51.9 μL, 5.5 mmol, 10 equiv) was added and shaken for 30 min. After it was washed by DCM (5 mL × 3) and DMF (5 mL × 3), a solution of 20% piperidine in 5 mL DMF was added to the above resin. The mixture was shaken for 1 hour at room temperature. Then the resin was washed by DCM (5 mL × 3) and DMF (5 mL × 3).

A mixture of Boc-Ile-OH (277.4 mg, 1.2 mmol, 4.0 equiv), PyBOP (624.5 mg, 1.2 mmol, 4.0 equiv) and DIEA (310.2 mg, 418.1 μL, 2.4 mmol, 8.0 equiv) in 5 mL DMF was added and shaken for 10 hours at room temperature. After the solvent was drained, 2 mL TFA was added and shaken for 5 min to remove the Boc group. Then the resin was washed by DCM (5 mL × 5) and DMF (5 mL × 5). The following amino acids were coupled through the general Boc-SPPS strategy.

Before peptide was cleaved from resin, it was treated with a mixture of TMSOTf/TFA/thioanisole (1:8.5:0.5, *v/v/v*) at 0 ^oC for 1 hour to remove the protective groups. Then the resin was washed by DCM (5 mL × 5). The resin was added into 5 mL DCM/TFA (95:5, *v/v*) at -78 ^oC and treated with O_3 for 5 min. Followed the addition of Me_2S, the solution was allowed to warm up and stirred at room temperature for another 1 hour. The reaction mixture was concentrated under low pressure and purified by preparative HPLC (20-60% CH_3CN/H_2O over 30 min) to afford compound **11** (150.7 mg, 0.18 mmol, 22.7% yield over all previous steps).[4] Compound **11** was monitored by LC-MS (ESI): calculated for $C_{43}H_{64}N_7O_{10}^+$ [M+H$^+$]: 838.5; found: 838.2 (**Supplementary Figure 33**).

Synthesis of linear hexapeptide salicylaldehyde (SAL) esters S7, S8, S9[4]

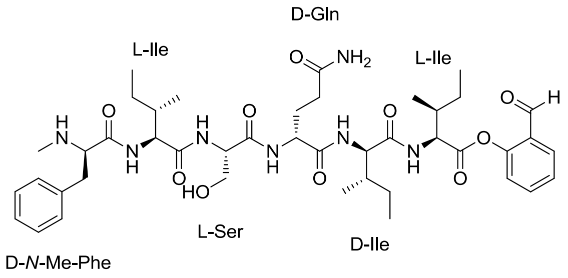

S7

Compound **S7** (D-*N*-Me-Phe-L-Ile-L-Ser-D-Gln-D-Ile-L-Ile-SAL ester) was synthesized through the same method as compound **11** (167.4 mg, 0.20 mmol, 36.3% yield over all steps).[4] Compound **S7** was monitored by LC-MS (ESI): calculated for $C_{43}H_{64}N_7O_{10}^+$ [M+H$^+$]: 838.5; found: 838.5 (**Supplementary Figure 34**).

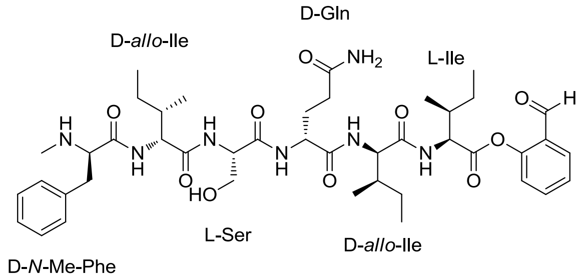

S8

Compound **S8** (D-*N*-Me-Phe-D-*allo*-Ile-L-Ser-D-Gln-D-*allo*-Ile-L-Ile-SAL ester) was synthesized through the same method as compound **11** (165.1 mg, 0.20 mmol, 36.3% yield over all steps).[4] Compound **S8** was monitored by LC-MS (ESI): calculated for $C_{43}H_{64}N_7O_{10}^+$ [M+H$^+$]: 838.5; found: 838.5 (**Supplementary Figure 35**).

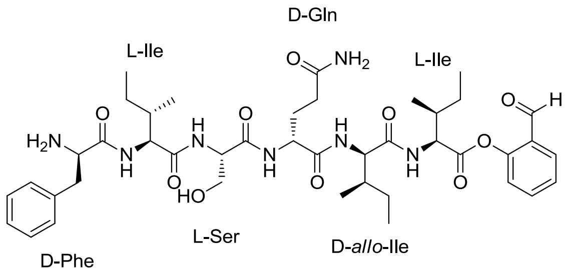

S9

Compound **S9** (D-Phe-L-Ile-L-Ser-D-Gln-D-*allo*-Ile-L-Ile-SAL ester) was synthesized through the same method as compound **11** (160.2 mg, 0.19 mmol, 34.8% yield over all steps).[4] Compound **S9** was monitored by LC-MS (ESI): calculated for $C_{42}H_{62}N_7O_{10}^+$ [M+H$^+$]: 824.5; found: 824.3 (**Supplementary Figure 36**).

Synthesis of Teixobacitin (12) by Serine ligation

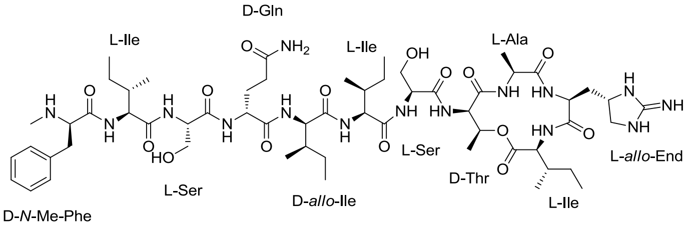

12

Compound **9** (2.0 mg, 3.8 μmol) and peptide SAL ester **11** (4.0 mg, 0.005 mmol, 1.5 equiv) was dissolved in a mixture of 400 μL pyridine/AcOH (mole/mole=6:1) and stirred at room temperature for 10 hours. After the solvent was removed by lyophilization, 1 mL TFA/H_2O/TIPS (*v/v/v*=94:5:1) was added and stirred for 1 hour. TFA/H_2O/TIPS was blown away by a condensed air stream. The residue was purified by preparative HPLC (20-60% CH_3CN/H_2O over 30 min) to afford compound **12** as a TFA salt (1.8 mg, 1.4 μmol, 36.8% yield). Then it was dissolved in CH_3CN/H_2O (*v/v*=1:1, containing 0.1% HCl) and lyophilizated for three times to afford compound **12** as an HCl salt. Compound **12** was monitored by LC-MS (ESI): calculated for $C_{58}H_{96}N_{15}O_{15}^{+}$ [M+H^{+}]: 1242.7; found: 1242.8 (**Supplementary Figure 38**).

Synthesis of Teixobacitin analogs (13-16) by Serine ligation

Compound **13-16** (TFA salt) was synthesized through the same method as compound **12**.

Teixobactin analog 13 (D-*allo*-Ile-OH was replaced by D-Ile-OH):

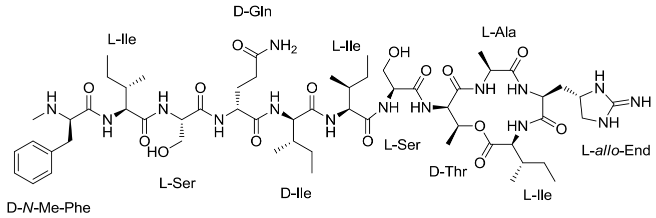

13

Compound **13** was monitored by LC-MS (ESI): calculated for $C_{58}H_{96}N_{15}O_{15}^{+}$ [M+H^{+}]: 1242.7; found: 1242.8 (**Supplementary Figure 40**).

Teixobactin analog 14 (L-*allo*-End-OH was replaced by L-Arg-OH; D-*allo*-Ile-OH was replaced by D-Ile-OH):

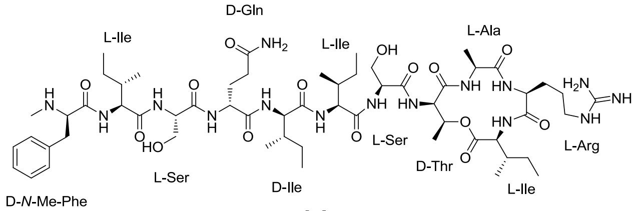

14

Compound **14** was monitored by LC-MS (ESI): calculated for $C_{58}H_{98}N_{15}O_{15}^{+}$ [M+H^{+}]: 1244.7; found: 1244.6 (**Supplementary Figure 41**).

Teixobactin analog 15 (D-Thr-OH was replaced by L-Thr-OH; L-*allo*-End-OH was replaced by L-Arg-OH; D-*allo*-Ile-OH was replaced by D-Ile-OH):

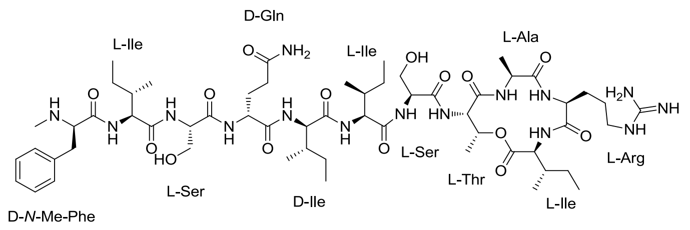

15

Compound **15** was monitored by LC-MS (ESI): calculated for $C_{58}H_{98}N_{15}O_{15}^{+}$ [M+H^{+}]: 1244.7; found: 1244.6 (**Supplementary Figure 42**).

Teixobactin analog 16 (L-*allo*-End-OH was replaced by L-Orn-OH):

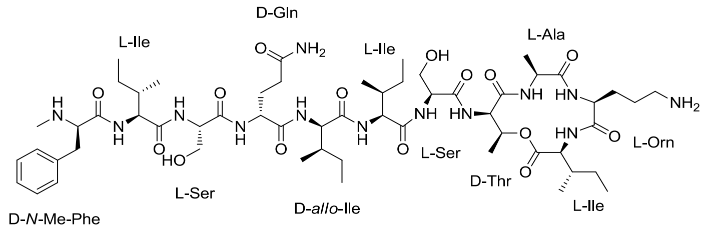

16

Compound **16** was monitored by LC-MS (ESI): calculated for $C_{57}H_{96}N_{13}O_{15}^{+}$ [M+H^{+}]: 1202.7; found: 1202.6 (**Supplementary Figure 43**).

Antibacterial activities:

Susceptibility to teixobactin and its analogues was tested on *Staphylococcus aureus* isolates using the standard broth dilution method as described by the Clinical and Laboratory Standards Institute[1,5] and MICs were determined according to CLSI guideline[6].

Supplementary references

1. L. L. Ling, T. Schneider, A. J. Peoples, *et al*. A new antibiotic kills pathogens without detectable resistance. *Nature*. **517**, 455-459 (2015).
2. C. William, C. Janet, R. David, T. Rondel, Y. Yu. A highly stereoselective and scalable synthesis of L-allo-Enduracididine. *Org. Lett*. **17**, 4620-4623 (2015).
3. H. Q. Lan, J. L. Ye, A. E. Wang, Y. P. Ruan, P. Q. Huang. A flexible asymmetric approach to Methyl 5-Alkyltetramates and its application in the synthesis of cytotoxic marine natural product Belamide A. *Chem. Eur. J*. **17**, 958-968 (2011).
4. C. T. T. Wong, H. Y. Lam, T. Song, G. H. Chen, X. C. Li. Synthesis of constrained Head-to-Tail cyclic tetrapeptides by an imine-induced ring-closing/contraction strategy. *Angew. Chem*. **125**, 10402-10405 (2013).
5. CLSI. *Methods for Dilution Antimicrobial Susceptibility Tests for Bacteria That Grow Aerobically; Approved Standard - Ninth Edition. CLSI document M07-A9, Wayne, PA: Clinical and Laboratory Standards Institute* (2012).
6. CLSI. *Performance Standards for Antimicrobial Susceptibility Testing；Twenty-sixth informational supplement. CLSI document M100-S26, Wayne, PA: Clinical and Laboratory Standards Institute* (2016).
